# TfOH mediated intermolecular electrocyclization for the synthesis of pyrazolines and its application in alkaloid synthesis†

**DOI:** 10.1039/c8ra05702h

**Published:** 2018-08-24

**Authors:** Sesuraj Babiola Annes, Pothiappan Vairaprakash, Subburethinam Ramesh

**Affiliations:** Department of Chemistry, School of Chemical and Biotechnology, SASTRA Deemed University Thanjavur Tamil Nadu India vairaprakash@scbt.sastra.edu ramesh_s@scbt.sastra.edu +91-4362-264120 +91-4362-264101-3614 +91-4362-264101-3791

## Abstract

TfOH mediated easy access to interesting pyrazolines starting from an aldehyde, phenylhydrazine and styrene has been developed. The scope of this synthetic methodology has been explored by synthesizing various 1,3,5-trisubstituted pyrazolines in very good yields with very high regioselectivity. The origin of regioselectivity has been explained by comparing the stability of possible intermediate carbocations. The synthetic utility of a green solvent has been explored by synthesizing some of pyrazolines in a DES medium. The synthetic application of the present methodology is employed in the synthesis of a pyrazoline alkaloid.

## Abstract 2

A metal free and green synthetic methodology employing aldehydes, phenylhydrazine and styrene mediated by TfOH has been developed to access 1,3,5-trisubstituted pyrazolines. The synthetic application of the methodology is demonstrated in the synthesis of a pyrazoline alkaloid.

## Graphical abstract

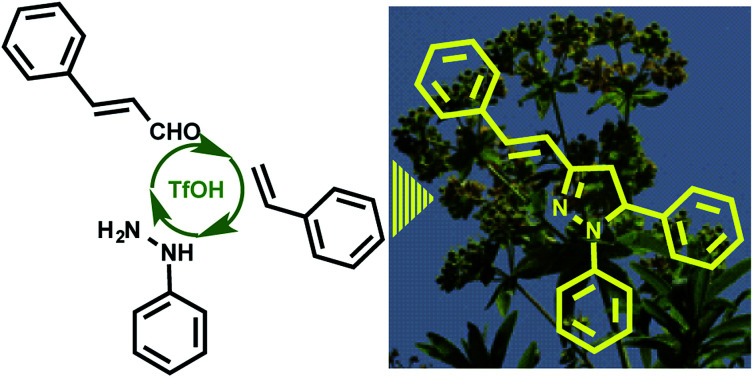

Pyrazoles and pyrazolines are known to exhibit interesting biological and photo-physical behaviours. The biological activities of pyrazoles/pyrazolines have been discussed in several reviews.^1–4^ A few representative examples of biologically and medicinally important pyrazoles and pyrazolines are given in Fig. 1.^5–7^ In particular, considerable interest has been focused on 1,3,5-trisubstituted pyrazoline derivatives due their potential pharmacological activities including (i) antitubercular activity against the H37Rv strain of Mycobacterium,^9^ (ii) antiproliferative activity,^8,10^ (iii) antibacterial activity,^11–13^ (iv) antiobesity effect in an animal model of the potent cannabinoid CB1 receptor antagonist,^14^ (v) pre-emergent herbicide activity against various kinds of weeds,^15^ and (vi) ACE-inhibitory activity with 0.123 mM IC504.^16^ Pyrazoline derivatives show enhanced biological activity compared with their corresponding pyrazoles.^17^ In addition, the pyrazoline motif is known to exhibit photo-luminescent behaviour due to intra-molecular charge transfer (ICT) in the excited state and also shows hole transport behaviour.^18–24^

**Fig. 1:**
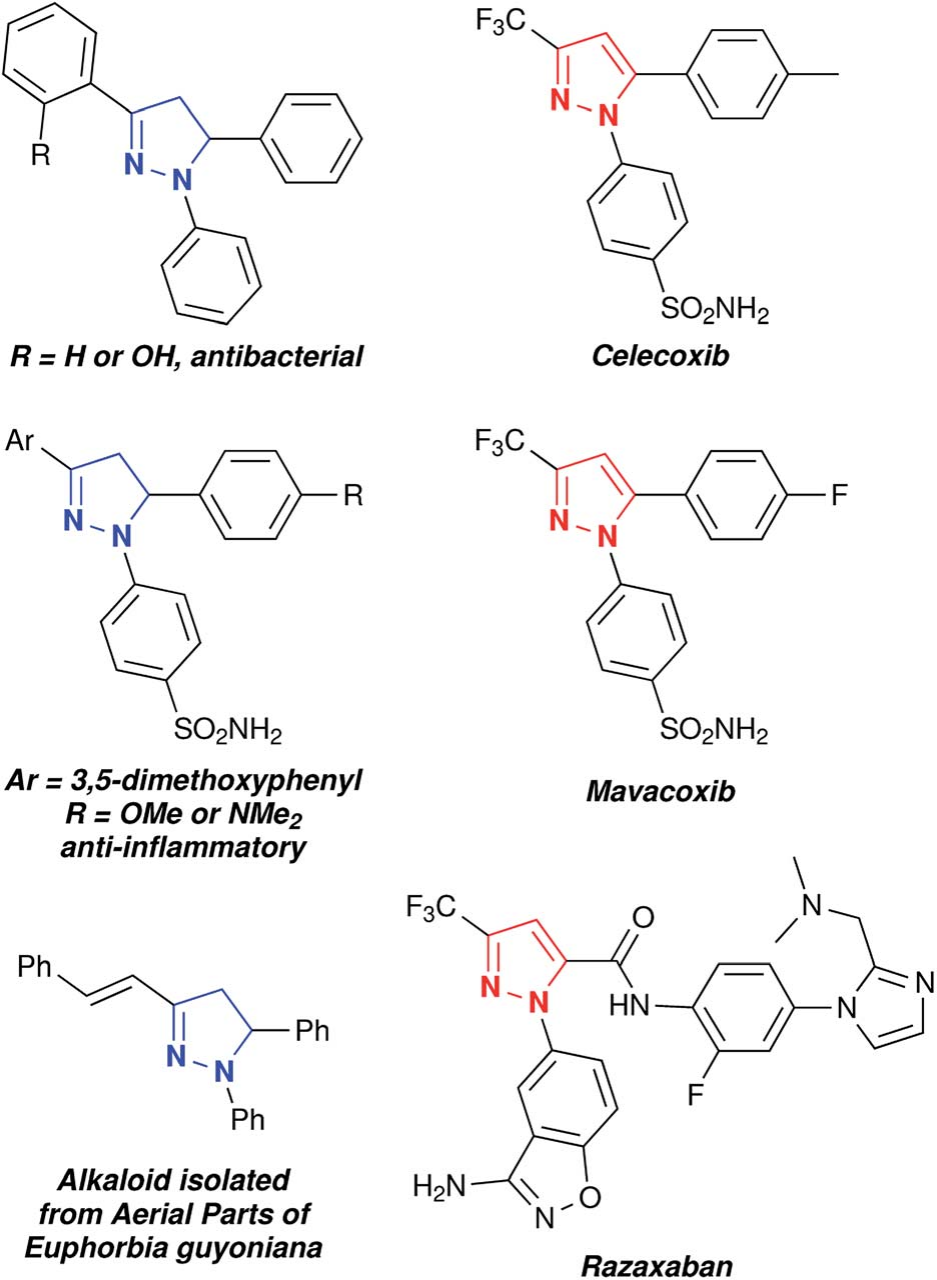
Representative examples of medicinally important 1,3,5-trisubstituted pyrazolines and pyrazoles.

Various synthetic approaches have been developed to access these biologically important 1,3,5-trisubstituted pyrazoline/pyrazole compounds.^25–28^ The most general synthetic approach proceeding *via* a reaction of 1,3-dicarbonyl compounds with arylhydrazines results in poor regioselectivity.^3,29,30^ A synthetic method which employs appropriate chalcones and arylhydrazines has been considered to be the most widely accepted method for accessing pyrazolines, but this method falls behind due to a greater number of synthetic steps involved.^31–34^ Recently Wang *et al.* disclosed a methodology proceeding *via* a three component [3 + 2] cycloaddition using 20 mol% Cu(OTf)_2_ at elevated temperature.^35^ The development of synthetic methodology to prepare active pharmaceutical ingredients (APIs) would preferably involve (i) high regioselectivity, (ii) diversity in substrates, (iii) the least number of synthetic steps involved and (iv) attaining target compounds, free of metal traces. Hence, a regioselective tandem one-pot intermolecular electrocyclization reaction under metal free condition to access 1,3,5-trisubstituted pyrazolines would be of great importance.

In this regard, we are disclosing a general, metal free and green synthetic methodology to access diverse pyrazoline derivatives with various functionalities including –NO_2_, –OH and aliphatic groups using arylhydrazines, aldehydes and styrenes. We have obtained the corresponding pyrazoline products in very good yields with enhanced regioselectivity. In addition, we have employed this synthetic methodology in the synthesis of a pyrazoline alkaloid (1,5-diphenyl-3-styryl-2-pyrazoline).

In our initial studies to attempt metal free conditions, iodine mediated intermolecular electrocyclization of tolualdehyde 1a, phenylhydrazine 2a and styrene 3 was explored (Table 1). The use of a substoichiometric amount of iodine (0.2 equiv.) yielded the corresponding pyrazoline 4a in 25% yield (Table 1, entry 1). Increasing the amount of iodine did not result in considerable enhancement of the yield (Table 1, entries 1–5). Due to it being less hazardous among the screened solvents, acetonitrile was used as a solvent for further screening of other non-metal catalysts including TfOH, PhIOAc, NaI, NBS, CAN, l-proline, CH(OMe)_3_, TFA, p-TSA and MeSO_3_H (Table 1, entries 6–13). TfOH was found to be the most effective in mediating electrocyclization and pyrazoline 4a was obtained in 82% yield (Table 1, entry 12). It is presumed that TfOH alone is capable of forming an intermediate carbocation I-1*via* hydride abstraction from hydrazone by the superacid species TfOH_2_^+^ (Scheme 1).^36,37^ The use of other solvents such as H_2_O, DMF, DMSO and ethanol did not result in product formation, as TfOH might be deactivated by these solvents (see ESI, Table S1†).

**Table 1:** Optimization of reaction conditions in the synthesis of pyrazoline 4a^a^

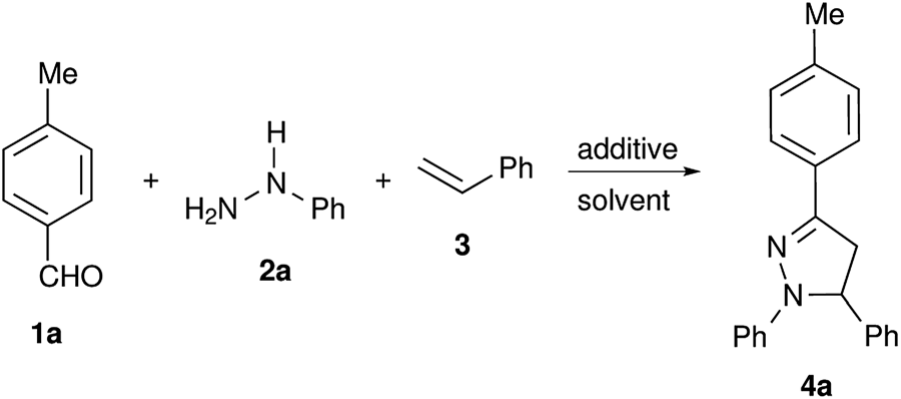				
Entry	Additive (equiv.)	Solvent	Time (h)	Yield^b^ (%)
1	I_2_ (0.2)	CH_3_CN	24	25
2	I_2_ (1.0)	CH_3_CN	24	34
3	I_2_ (1.0)	Toluene	24	40
4	I_2_ (1.0)	H_2_O	24	35
5	I_2_ (1.0)	EtOAc	24	Trace
6	PhI(OAc)_2_ (0.2)	CH_3_CN	24	ND
7	NaI (1.0)	CH_3_CN	24	ND
8	NBS (1.0)	CH_3_CN	24	ND
9	CAN (1.0)	CH_3_CN	24	ND
10	l-Proline (0.3)	CH_3_CN	24	ND
11	CH(OMe)_3_ (1.0)	CH_3_CN	24	ND
**12**	**TfOH (1.0)**	**CH**_**3**_ **CN**	**7**	**82**
13	TFA (1.0)	CH_3_CN	24	ND
14	p-TSA (1.0)	CH_3_CN	24	18
15	MeSO_3_H (1.0)	CH_3_CN	24	13

a A solution of tolualdehyde 1a (1.0 mmol) and phenylhydrazine 2a (1.0 mmol) in solvent (1.0 mL) was treated with additive followed by styrene 3 (1.0 mmol) and stirred.

b Isolated yield; ND = not detected.

**Scheme 1:**
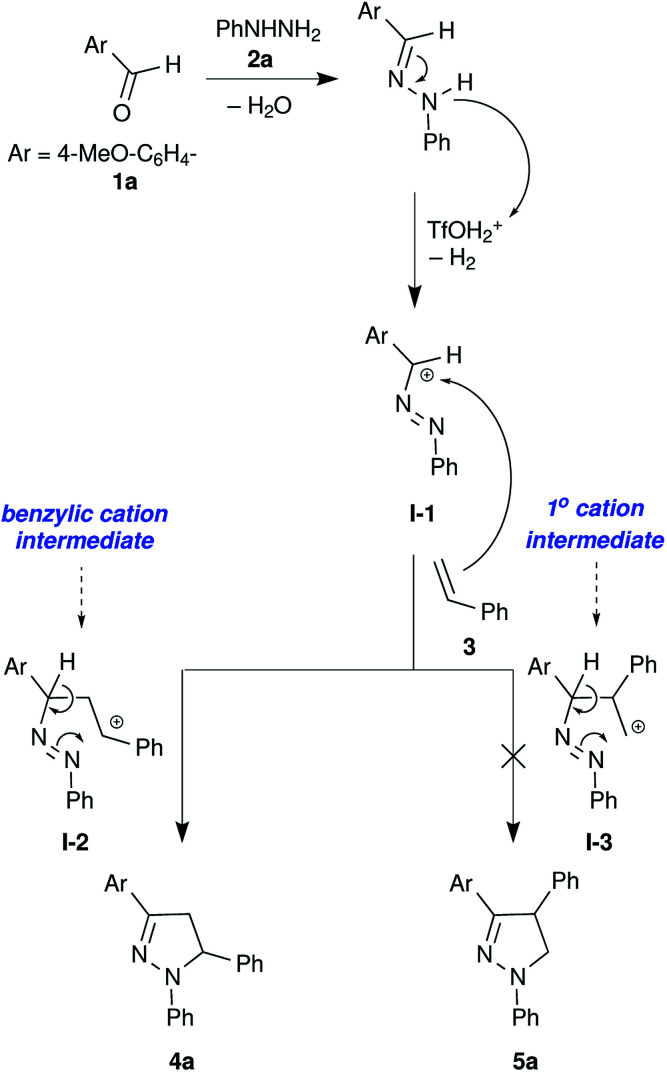
Origin of regioselectivity in the formation of pyrazoline 4a.

We have observed very good regioselectivity in the formation of pyrazolines mediated by TfOH. Mechanistically, the carbocation I-1 can interact with either of the alkenyl carbons in styrene resulting in two types of cationic intermediates: (i) the most stable benzylic carbocation I-2 and (ii) the least stable primary carbocation I-3. The stability of the benzylic carbocation I-2 over the primary carbocation I-3 directs the reaction pathway towards the formation of a 5-phenyl substituted product 4a over a 4-phenyl substituted product 5a (Scheme 1).

The scope of this synthetic methodology has been studied by varying the substrates. Both aromatic and aliphatic aldehydes reacted well under the reaction conditions and their corresponding pyrazolines were obtained in yields of up to 86% (Table 2). The yields were in accordance with the stability of the corresponding benzylic cations I-1. In the reactions employing aromatic aldehydes with electron donating groups, pyrazolines were obtained in very good yields (Table 2, entries 1 and 2), as I-1 might receive additional stability from electron donating substituents. The methoxy group in the *meta* position did not play a considerable role in stabilizing the benzylic carbocation I-1, hence the yield of the corresponding pyrazoline 4c was moderate (Table 2, entry 3). Analogous to this, products were obtained in moderate yields for aldehydes with halogen substitution (Cl and Br) and without any substitution, as there is neutrality between the electron withdrawing effect through –I and the electron releasing effect through +R in H/Cl/Br– substituted aldehydes (2, entries 4–6). The high electronegativity of fluorine destabilizes I-1, hence its corresponding pyrazoline 4g was obtained in low yield (Table 2, entry 7). Similarly, pyrazolines 4h and 4i were obtained in poor yields due to the strong electron withdrawing effect of the –NO_2_ group *via* –I and –R (Table 2, entries 8 and 9). In every case, the obtained regioselectivity was very high and benzylic cation intermediate I-2 derived compounds were obtained as the only products.

**Table 2:** Synthesis of various pyrazolines 4a–m^a^

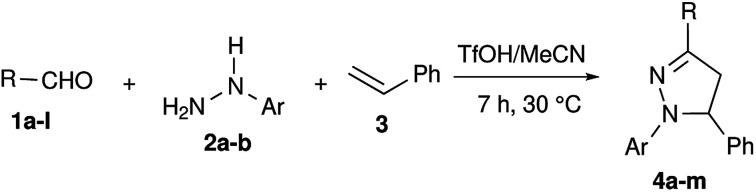				
Entry	R =	Ar =	Product	Yield^b^ (%)
1	4-Me–C_6_H_4_– 1a	C_6_H_5_– 2a	4a	82
2	4-MeO–C_6_H_4_– 1b	C_6_H_5_– 2a	4b	73
3	3-MeO–C_6_H_4_– 1c	C_6_H_5_– 2a	4c	57
4	C_6_H_5_– 1d	C_6_H_5_– 2a	4d	63
5	4-Br–C_6_H_4_– 1e	C_6_H_5_– 2a	4e	61
6	4-Cl–C_6_H_4_– 1f	C_6_H_5_– 2a	4f	58
7	4-F–C_6_H_4_– 1g	C_6_H_5_– 2a	4g	39
8	4-NO_2_–C_6_H_4_– 1h	C_6_H_5_– 2a	4h	24
9	2-NO_2_–C_6_H_4_– 1i	C_6_H_5_– 2a	4i	45
10	2-HO–C_6_H_4_– 1j	C_6_H_5_– 2a	4j	82
11	CH_3_CH_2_CH_2_– 1k	C_6_H_5_– 2a	4k	86
12	(CH_3_)_2_CH– 1l	C_6_H_5_– 2a	4l	58
13	4-Me–C_6_H_4_– 1a	4-Me–C_6_H_4_– 2b	4m	82

a A solution of aldehyde 1a–l (1.0 mmol) and arylhydrazine 2a, 2b (1.0 mmol) in CH_3_CN (1.0 mL) was treated with TfOH followed by styrene 3 (1.0 mmol) and stirred.

b Isolated yield.

Using this methodology, we have attempted the synthesis of bis-pyrazoline 6 from terephthalaldehyde 1m. The bis-pyrazoline 6 was obtained in very low yield, which is also in accordance with the proposed mechanism depicted in Scheme 1. The formation bis-pyrazoline 6 was expected to proceed *via* a bis-benzylic cation I-4 (Scheme 2), which is highly destabilized by conjugation between two cationic sites. Thus, formation of the bis-benzylic cation I-4 is less probable and resulted in the observed poor yield of bis-pyrazoline 6 (18%) even after 48 h of reaction time. Bis-pyrazoline 6 was structurally characterized by NMR spectroscopy and high resolution mass spectrometry (HRMS) analysis.

**Scheme 2:**
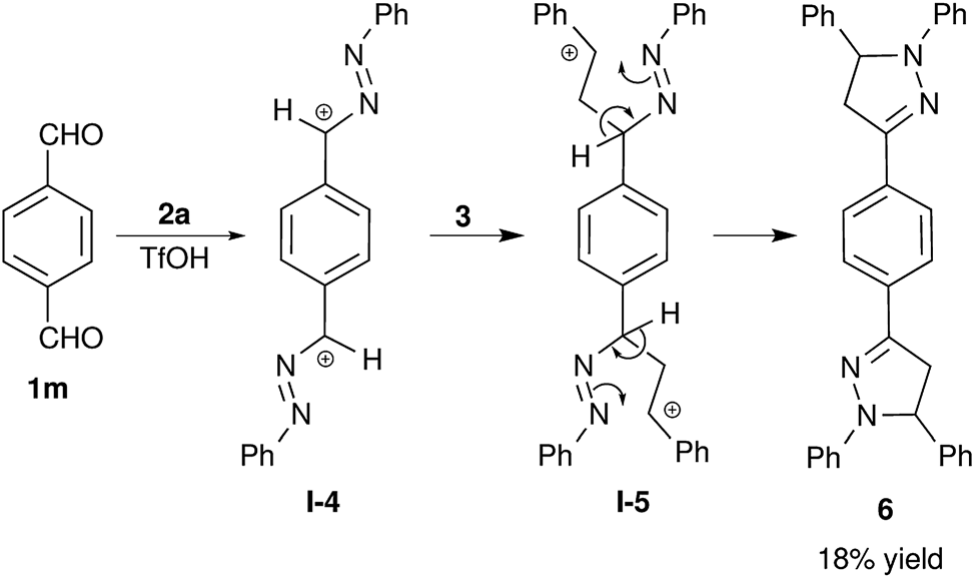
Synthesis of bis-pyrazoline 7 from terephthalaldehyde.

We wanted to apply this synthetic methodology in the synthesis of a pyrazoline alkaloid. Many species of the genus Euphorbia are medicinally important and known to treat various ailments including gonorrhea, skin diseases, gastrointestinal disorders, migraines and anaphylaxis.^38–41^ Alkaloid 7 (1,5-diphenyl-3-styryl-2-pyrazoline) was isolated from aerial parts of Euphorbia guyoniana.^42^ We have adopted TfOH mediated electrocyclization to access alkaloid 7 starting from cinnamaldehyde 1n, phenylhydrazine 2a and styrene 3. In this reaction, the formation of pyrazole 8*via* intramolecular electrocyclization is expected to compete with the formation of the desired alkaloid 7*via* intermolecular electrocyclization (Scheme 3). Interestingly, the reaction with cinnamaldehyde did not undergo intramolecular cyclization and the required alkaloid 7 was obtained in 82% yield.

**Scheme 3:**
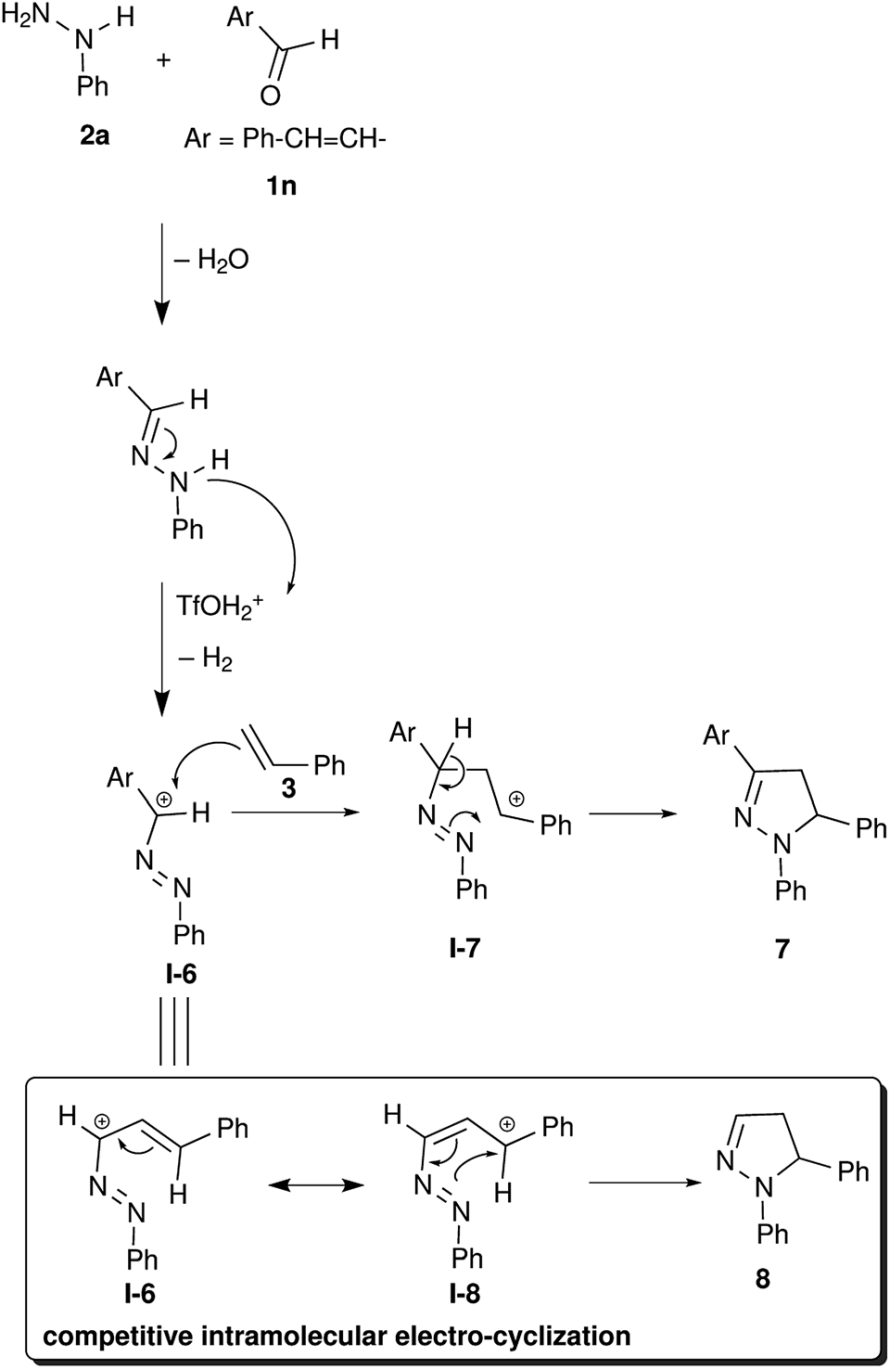
Application in alkaloid synthesis: synthesis of alkaloid 7.

Recently, deep eutectic solvents (DES) have been used as green solvents in various applications.^43–45^ We have explored the use of a few DES including the eutectic mixture of (i) ChCl : urea, (ii) ChCl : PTSA, (iii) ChCl : TfOH and (iv) ChCl : glycol in our synthetic methodology to access trisubstituted pyrazolines (see ESI, Table S2†). The eutectic mixture ChCl : PTSA was found to be effective among the DES screened and corresponding 1,3,5-trisubstituted pyrazolines were obtained in yields of up to 61%, without the use of any additives (Table 3). We believe that the ChCl : PTSA DES itself can induce the formation of intermediate I-1 to some extent and can result in the formation of pyrazolines, even in the absence of catalysts.

**Table 3:** Synthesis of various pyrazolines in DES media^a^

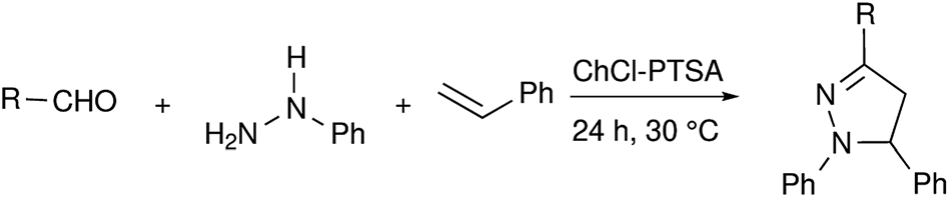				
Entry	R =	Ar =	Product	Yield^b^ (%)
1	4-Me–C_6_H_4_– 1a	C_6_H_5_– 2a	4a	45
2	2-HO–C_6_H_4_– 1j	C_6_H_5_– 2a	4j	61
3	CH_3_CH_2_CH_2_– 1k	C_6_H_5_– 2a	4k	45
4	C_6_H_5_–CH=CH– 1n	4-Me–C_6_H_4_– 2b	Alkaloid 7	54

a A mixture of aldehyde (1.0 mmol) arylhydrazine (1.0 mmol) and styrene (1 mmol) in DES (1.0 mL) stirred at 30 °C for 24 h.

b Isolated yield.

In conclusion, we have developed a metal free TfOH or DES mediated intermolecular electrocyclization of aldehydes, hydrazine and styrene to generate 1,3,5-trisubstituted pyrazolines. The observed very high regioselectivity of the reaction has been rationalized by the stability of the proposed benzylic cation intermediate. The substrate scope has been studied and various pyrazolines have been obtained in moderate to very good yields. The obtained yields were in accordance with the stability of the proposed intermediate carbocations obtained by the electronic effects of substitutions. Working towards greener synthesis, we have screened various deep eutectic mixtures as media for this transformation. The DES ChCl : PTSA was found to be effective in mediating pyrazoline formation and the corresponding pyrazolines were obtained in moderate yields in the absence of any other catalysts. The application of this synthetic methodology has been demonstrated in the synthesis of a natural product, alkaloid 7 obtained from aerial parts of Euphorbia guyoniana.

## Conflicts of interest

There are no conflicts to declare.

## Supplementary Material

RA-008-C8RA05702H-s001
