# Supplementary material for: TfOH mediated intermolecular electrocyclization for the synthesis of pyrazolines and its application in alkaloid synthesis
Source: RSC Adv. 2018 Aug 24;8(53):30071–5. doi: 10.1039/c8ra05702h (PMC9085413; doi:10.1039/c8ra05702h)
Supplement: RA-008-C8RA05702H-s001 [file RA-008-C8RA05702H-s001.pdf]

Electronic Supplementary Material (ESI) for RSC Advances.
This journal is © The Royal Society of Chemistry 2018

TfOH mediated intermolecular electrocyclization for the synthesis of pyrazolines and application in alkaloid synthesis

Babiola Annes, Pothiappan Vairaprakash* and Subburethinam Ramesh*

Department of Chemistry, School of Chemical and Biotechnology,

SASTRA Deemed University, Thanjavur, Tamil Nadu, India 613 4001

E-mail: vairaprakash@scbt.sastra.edu; ramesh_s@scbt.sastra.edu

General Procedure for the synthesis of pyrazoline

Into the reaction mixture of phenylhydrazine (1.0 mmol) and acetonitrile (10 V) were added aldehyde (1.0 mmol). The reaction mixture then was stirred for 10 min. at 30 ^{o}C followed by TfOH (1.0 mmol) and styrene (1.0 mmol) were added. The mixture was stirred at the same temperature till completion of the reaction which was monitored by TLC. After completion of the reaction, the crude was quenched by sat. solution of sodiumbicarbonate and extracted with ethylacetate. The organic layer was separated and dried with anhydrous sodium sulphate and then concentrated under reduced pressure to get crude compound. The pyrazoline was purified by 60-120 silica get column chromatography using hexane/ethylacetate as eluents.

1,5-diphenyl-3-(p-tolyl)-4,5-dihydro-1H-pyrazole (4a)

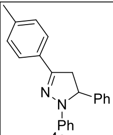
4a

M.P = 159-161 ^{o}C, ^{1}H NMR (400 MHz, $CDCl_3$, δ ppm) 7.66-7.64 (2H, m), 7.37-7.36 (4H, m), 7.31-7.28 (1H, m), 7.24-7.19 (4H, m), 7.11-7.09 (2H, m), 6.83-6.79 (1H, m), 5.28 (1H, dd, J = 7.3 Hz & 12.4 Hz), 3.85 (1H, dd, J = 12.4 Hz & J = 17.1 Hz), 3.16 (1H, dd, J = 7.3 Hz & 17.1 Hz), 2.41 (3H, s). The spectral data showed good agreement with the literature data.[1]

3-(4-methoxyphenyl)-1,5-diphenyl-4,5-dihydro-1H-pyrazole (4b)

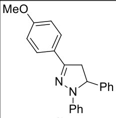
4b

M.P = 148 -150 ^{o}C, ^{1}H NMR (400 MHz, $CDCl_3$, δ ppm) 7.71-7.67 (2H, m), 7.36-7.35 (4H, m), 7.30-7.29 (1H, m), 7.22-7.17 (2H, m), 7.09-7.06 (2H, m), 6.96-6.92 (2H, m), 6.78 (1H, tt, J = 1.08 Hz & 7.3Hz), 5.25 (1H, dd, J = 7.3 Hz & 12.4 Hz), 3.88-3.81 (4H, m), 3.14 (1H, dd, J = 7.4 Hz & J = 17.0 Hz).
The spectral data showed good agreement with the literature data.[2]

3-(3-methoxyphenyl)-1,5-diphenyl-4,5-dihydro-1H-pyrazole (4c)

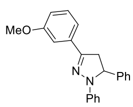
4c

M.P = 108-110 ^{o}C, ^{1}H NMR (300 MHz, $CDCl_3$, δ ppm) 7.33-7.22 (8H, m), 7.20-7.15 (2H, m), 7.08-7.06 (2H, m), 6.90-6.86 (1H, m), 6.78 (1H, t, J = 7.2 Hz), 5.27 (1H, dd, J = 7.2 Hz & 12.4 Hz), 3.88 – 3.76 (4H, m), 3.13 (1H, dd, J = 7.2 Hz & J = 17.1 Hz). ^{13}C NMR (75 MHz, $CDCl_3$, δ ppm): 159.8, 146.6, 144.8, 142.6, 134.1, 129.6, 129.2, 128.9, 127.6, 125.9, 119.2, 118.5, 114.8, 113.4, 110.6, 64.6, 55.4, 43.7.

1,3,5-triphenyl-4,5-dihydro-1H-pyrazole (4d)

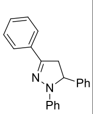
4d

M.P = 138 – 140 ^{o}C, ^{1}H NMR (300 MHz, $CDCl_3$, δ ppm) 7.74-7.71 (2H, m), 7.41-7.22 (8H, m), 7.20-7.15 (2H, m), 7.08-7.06 (2H, m), 6.78 (1H, t, J = 7.2 Hz), 5.27 (1H, dd, J = 7.3 Hz & 12.4 Hz), 3.83 (1H, dd, J = 12.4 Hz & 17.1 Hz), 3.14 (1H, dd, J = 7.3 Hz & J = 17.1 Hz).
The spectral data showed good agreement with the literature data.[1]

3-(4-bromophenyl)-1,5-diphenyl-4,5-dihydro-1H-pyrazole (4e)

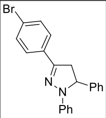
4e

M.P = ^{o}C, ^{1}H NMR (500 MHz, $CDCl_3$, δ ppm) 7.61-7.59 (2H, m), 7.53-7.52 (2H, m), 7.38-7.28 (5H, m), 7.23-7.20 (2H, m), 7.11-7.09 (2H, m), 6.85-6.82 (1H, m), 5.31 (1H, dd, J = 5.4 Hz & 10.6 Hz), 3.83 (1H, dd, J = 16.8 Hz & 12.5 Hz), 3.12 (1H, dd, J = 7.3 Hz & J = 17.0 Hz).The spectral data showed good agreement with the literature data.[2]

3-(4-chlorophenyl)-1,5-diphenyl-4,5-dihydro-1H-pyrazole (4f)

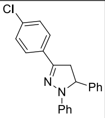
4f

MP – 156-158 ^{o}C, ^{1}H NMR (300 MHz, $CDCl_3$, δ ppm) 7.65 -7.61 (2H, m), 7.36 - 7.25 (7H, m), 7.20-7.15 (2H, m), 7.07-7.04 (2H, m), 6.79 (1H, t, J = 7.2 Hz), 5.28 (1H, dd, J = 7.3 Hz & 12.4 Hz), 3.80 (1H, dd, J = 12.4 Hz & J = 17.1 Hz), 3.10 (1H, dd, J = 7.3 Hz & 17.1 Hz). The spectral data showed good agreement with the literature data.[2]

3-(4-fluorophenyl)-1,5-diphenyl-4,5-dihydro-1H-pyrazole (4g)

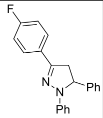
4g

M.P = 138-140 ^{o}C, ^{1}H NMR (500 MHz, $CDCl_3$, δ ppm) 7.74-7.71 (2H, m), 7.37-7.34 (4H, m), 7.32-7.28 (1H, m), 7.23-7.19 (2H, m), 7.12-7.08 (4H, m), 6.83-6.80 (1H, m), 5.30 (1H, dd, J = 4 Hz & 12.4 Hz), 3.84 (1H, dd, J = 12.4 Hz & 17 Hz), 3.15 (1H, dd, J = 7.3 Hz & J = 17.0 Hz). The spectral data showed good agreement with the literature data.[1]

3-(4-nitrophenyl)-1,5-diphenyl-4,5-dihydro-1H-pyrazole (4h)

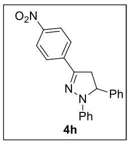

MP – 188-190 ^{o}C, ^{1}H NMR (300 MHz, $CDCl_3$, δ ppm) 8.25 -8.22 (2H, m), 7.83-7.80 (2H, m), 7.38-7.28 (5H, m), 7.23-7.18 (2H, m), 7.12-7.09 (2H, m), 6.85 (1H, t, J = 7.2 Hz); 5.42 (1H, dd, J = 7.0 Hz & 12.3 Hz), 3.87 (1H, dd, J = 12.7 & J = 17.1 Hz), 3.16 (1H, dd, J = 7.0 Hz & 17.1 Hz); ^{13}C NMR (75 MHz, $CDCl_3$, δ ppm): 42.8, 64.9, 113.8, 120.3, 124.0, 125.7, 125.8, 127.9, 129.1, 129.3, 139.0, 141.7, 143.6, 143.9, 147.0.

The spectral data showed good agreement with the literature data.[4]

3-(2-nitrophenyl)-1,5-diphenyl-4,5-dihydro-1H-pyrazole (4i)

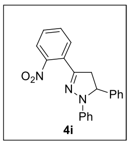

MP – 148-150 ^{o}C, ^{1}H NMR (300 MHz, $CDCl_3$, δ ppm) 7.69-7.66 (1H, m), 7.61-7.52 (2H, m), 7.45-7.39 (1H, m), 7.37-7.24 (5H, m), 7.19-7.14 (2H, m), 7.01-6.90 (2H, m), 6.81 (1H, t, J = 7.3 Hz); 5.32 (1H, dd, J = 7.3 Hz & 12.4 Hz), 3.78 (1H, dd, J = 12.4 & J = 17.0 Hz), 3.05 (1H, dd, J = 7.3 Hz & 17.0 Hz)

2-(1,5-diphenyl-4,5-dihydro-1H-pyrazol-3-yl)phenol (4j)

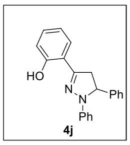

M.P = 178 -180 ^{o}C, ^{1}H NMR (400 MHz, $CDCl_3$, δ ppm) 10.83 (1H, S), 7.39-7.35 (4H, m), 7.34-7.28 (2H, m), 7.28-7.21 (2H, m), 7.16-7.08 (2H, m), 7.00-6.98 (2H, m), 6.93-6.85 (2H, m), 5.28-5.23 (1H, dd, J = 12.3 Hz & 7.5 Hz), 4.02-3.94 (1H, dd, J = 12.3 Hz & 17.2 Hz), 3.31-3.25 (1H, dd, 17.2 Hz & 7.56 Hz)

The spectral data showed good agreement with the literature data.[1]

1,5-diphenyl-3-propyl-4,5-dihydro-1H-pyrazole (4k)

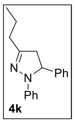

Viscous Liquid, ^{1}H NMR (300 MHz, $CDCl_3$, δ ppm) 7.36 -7.25 (5H, m), 7.18 - 7.11 (2H, m), 6.94-6.91 (2H, m), 6.73 (1H, t, J = 7.3 Hz), 4.99 (1H, dd, J = 8.0 Hz & 11.9 Hz), 3.40 (1H, dd, J = 11.9 Hz & J = 17.4 Hz), 2.70 (1H, dd, J = 8.0 Hz & 17.4 Hz), 2.37 (2H, t, J = 7.4 Hz), 1.68-1.56 (2H, m), 0.98 (3H, t, J = 7.4 Hz); ^{13}C NMR (75 MHz, $CDCl_3$, δ ppm): 152.1, 146.2, 143.2, 129.1, 128.9, 127.4, 125.9, 118.6, 113.2, 77.5, 77.1, 76.7, 64.5, 46.2, 32.2, 20.2, 13.9.

3-isobutyl-1,5-diphenyl-4,5-dihydro-1H-pyrazole (4l)

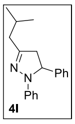

Viscous liquid, ^{1}H NMR (300 MHz, $CDCl_3$, δ ppm) 7.36 -7.27 (5H, m), 7.16 - 7.11 (2H, m), 6.94-6.91 (2H, m), 6.73 (1H, t, J = 7.3 Hz), 5.00 (1H, dd, J = 7.8 Hz & 11.9 Hz), 3.39 (1H, dd, J = 11.9 Hz & J = 17.5 Hz), 2.69 (1H, dd, J = 7.8 Hz & 17.5 Hz), 2.28 (2H, d, J = 7.3 Hz), 1.99-1.85 (1H, m), 0.98-0.95 (6H, m). ^{13}C NMR (100 MHz, $CDCl_3$, δ ppm): 151.4, 146.1, 143.2, 129.1, 128.9, 127.4, 125.9, 118.6, 113.2, 64.5, 46.6, 39.2, 26.5, 22.7, 22.5.

5-phenyl-1,3-di-p-tolyl-4,5-dihydro-1H-pyrazole (4m)

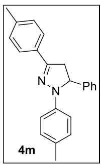

MP –162-164 ^{o}C, ^{1}H NMR (300 MHz, $CDCl_3$, δ ppm) 7.60 (2H, d, J = 8.1 Hz), 7.33-7.32 (4H, m), 7.29-7.25 (1H, m), 7.18 (2H, d, J = 8.1 Hz), 6.96-6.97 (4H, m), 5.20 (1H, dd, J = 7.7 Hz & 12.3 Hz), 3.80 (1H, dd, J = 12.3 & J = 17.0 Hz), 3.10 (1H, dd, J = 7.7 Hz & 17.0 Hz), 2.37 (3H, s), 2.22 (3H, s). ^{13}C NMR (75 MHz, $CDCl_3$, δ ppm): 143.1, 142.9, 138.5, 130.1, 129.4, 129.3, 129.1, 128.2, 127.5, 126.0, 125.7, 113.5, 64.9, 43.7, 21.4, 20.5.

Alkaloid 7

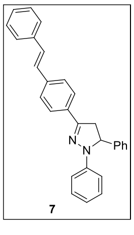

^{1}H NMR (300 MHz, $CDCl_3$, δ ppm) 7.46 -7.43 (2H, m), 7.36 - 7.32 (5H, m), 7.30-7.26 (3H, m), 7.20-7.13 (3H, m), 7.02-6.99 (2H, m), 6.81-6.76 (1H, m), 6.54 (1H, d, J = 16.3 Hz), 5.26 (1H, dd, J = 6.8 Hz & 12.4Hz), 3.72 (1H, dd, J = 12.3 Hz & J = 16.8 Hz), 3.02 (1H, dd, J = 6.8 Hz & 16.8 Hz)

The spectral data showed good agreement with the literature data.[3,4]

1,4-bis(1,5-diphenyl-4,5-dihydro-1H-pyrazol-3-yl)benzene (6)

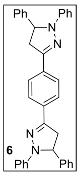

MP – 198-200 °C, ^{1}H NMR (300 MHz, $CDCl_3$, δ ppm) 7.71 (4H, s), 7.37-7.31 (10H, m), 7.21-7.16 (4H, m), 7.09-7.07 (4H, m), 6.79 (1H, t, J = 7.2 Hz); 5.29 (1H, dd, J = 7.2 Hz & 12.4 Hz), 3.86 (1H, dd, J = 12.4 & J = 17.1 Hz), 3.15 (1H, dd, J = 7.2 Hz & 17.1 Hz). HRMS [M+H] calculated 519.2549. Found 519.2526

Due to poor solubility either in DMSO-d_6 as well as in $CDCl_3$, we were unable to take ^{13}C NMR spectra.

Table 1. Complete optimization for the pyrazoline under metal free conditions

S. No	Reagent	Solvent	Temp. (°C)/Time	Yield (%)
1	I_2 (20 mol%)	CH_3CN	25-30 / 24	25
2	I_2 (1.0 equiv.)	CH_3CN	25-30 / 24	34
3	I_2 (1.0 equiv.)	Toluene	25-30 / 24	47
**4**	**I_2 (1.0 equiv.)**	**H_2O**	**25-30 / 24**	**35**
5	I_2 (1.0 equiv.)	Ethylacetate	25-30 / 24	Trace
6	$PhI(OAc)_2$ (20 mol%)	CH_3CN	25-30 / 24	ND
**7**	**TfOH (1.0 equiv.)**	**CH_3CN**	**25-30 / 7**	**82**
8	NaI (1.0 equiv.)	CH_3CN	25-30 / 24	ND
9	NBS (1.0 equiv.)	CH_3CN	25-30 / 24	ND
10	CAN (1.0 equiv.)	CH_3CN	25-30 / 24	ND
11	L-Proline(30 mol%)	CH_3CN	25-30 / 24	ND
12	$CH(OMe)_3$ (1.0 equiv.)	CH_3CN	25-30 / 24	ND
13	TfOH (1.0 equiv.)	H_2O	25-30 / 24	trace
14	TfOH (1.0 equiv.)	DCM	25-30 / 24	trace
15	TfOH (1.0 equiv.)	DMF	25-30 / 24	trace
16	TfOH (1.0 equiv.)	DMSO	25-30 / 24	trace
17	TfOH (1.0 equiv.)	Ethanol	25-30 / 24	ND
18	-	TFA	25-30 / 7	49
19	-	Acetic acid	25-30 / 24	ND

[a] Isolated Yield

Table 2. Complete optimization in DES medium

S.NO	SM 1	SM 2	Eutectic Mixture	Catalyst	Temperature	Yield%
1	$PhNHNH_2$. HCl	$PhCH_3CHO$	Ch.Chloride+ Urea	-	75 – 80 ^{0}C	Trace
2	$PhNHNH_2$	$PhCH_3CHO$	Ch.Chloride+ Glycerol	-	75 – 80 ^{0}C	Trace
3	$PhNHNH_2$	$PhCH_3CHO$	Ch.Chloride+ Urea	-	RT	Trace
4	$PhNHNH_2$	$PhCH_3CHO$	Ch.Chloride+ Urea	I_2(0.2 equiv)	RT	Trace
5	$PhNHNH_2$	$PhCH_3CHO$	Ch.Chloride+ Urea	NBS	50 ^{0}C	ND
6	$PhNHNH_2$	$PhCH_3CHO$	Ch.Chloride+ Urea	I_2(2 equiv)	50 ^{0}C	Trace
7	$PhNHNH_2$	$PhCH_3CHO$	Ethylene Glycol	TfOH	RT	Trace
8	$PhNHNH_2$	$PhCH_3CHO$	Ch.Chloride+ Ethylene Glycol	-	RT	Trace
9	$PhNHNH_2$	$PhCH_3CHO$	Ch.Chloride+ Ethylene Glycol	-	90 ^{0}C	Trace
10	$PhNHNH_2$	$PhCH_3CHO$	Ch.Chloride+ Ethylene Glycol	I_2(1 equiv)	RT	Trace
11	$PhNHNH_2$	$PhCH_3CHO$	Ch.Chloride+ Ethylene Glycol	TfOH(0.2 equiv)	RT	ND
12	$PhNHNH_2$	$PhCH_3CHO$	Ch.Chloride+ Ethylene Glycol	KOt(Bu) (1 equiv)	50 ^{0}C	Trace
13	$PhNHNH_2$	$PhCH_3CHO$	Ch.Chloride+ pTsOH	-	RT	35%
14	$PhNHNH_2$	$PhCH_3CHO$	CH_3CN+ pTsOH	-	RT	62%
15	$PhNHNH_2$	$PhCH_3CHO$	Ch.Chloride+ pTsOH(10 V)	-	RT	45%
16	$PhNHNH_2$	$PhCH_3CHO$	Ethanol	Ch.Chloride+ pTsOH(1 equiv)	RT	41%(1g of Silica 60-120 mesh added to the reaction)
17	$PhNHNH_2$	$PhCH_3CHO$	Ethanol	Ch.Chloride+ pTsOH(0.2 equiv)	RT ,70 ^{0}C	Trace
18	$PhNHNH_2$	$PhCH_3CHO$	Ch.Chloride+ Urea	pTsOH	RT ,70 ^{0}C	Trace
19	$PhNHNH_2$	$PhCH_3CHO$	Ethanol , Ch.Chloride+ pTsOH	-	RT	Trace
20	$PhNHNH_2$	$PhCH_3CHO$	Ch.Chloride+ Urea, Ch.Chloride+ pTsOH	-	RT	ND
21	$PhNHNH_2$	$PhCH_3CHO$	Ch.Chloride+ TfOH, CH_3CN(0.1 ml)	-	N_2 atm , RT ,70 ^{0}C	54%
22	$PhNHNH_2$	$PhCH_3CHO$	CH_3CN(1 ml)	Ch.Chloride+	N_2 atm ,	30%

				TfOH(1 equiv)	RT	
23	$PhNHNH_2$	$PhCH_3CHO$	Ethanol	TfOH	50 ^0C,70 ^0C	Trace
24	$PhNHNH_2$	$PhCH_3CHO$	Ch.Chloride+ pTsOH	-	RT	55%(Styrene 5 Equiv)

References

1. X. Wang, Y. M. Pan, X. C. Huang, Z. Y. Mao, H. S. Wang, *Org. Biomol. Chem.* **2014**, *12*, 2028-2032.

2. Q. Wu, P. Liu, Y. M. Pan, Y. L. Xu, H. S. Wang, *RSC Advances*, **2012**, *2*, 10167–10170.

3. P. Kundu, D. Banerjee, G. Maiti, N. Chattopadhyay, *Phys. Chem. Chem. Phys.* **2017**, *19*, 11937–11946.

4. G. S. Ananthnag, A. Adhikari, M. S. Balakrishna, *Catalysis Communication*, **2014**, *43*, 240-243.

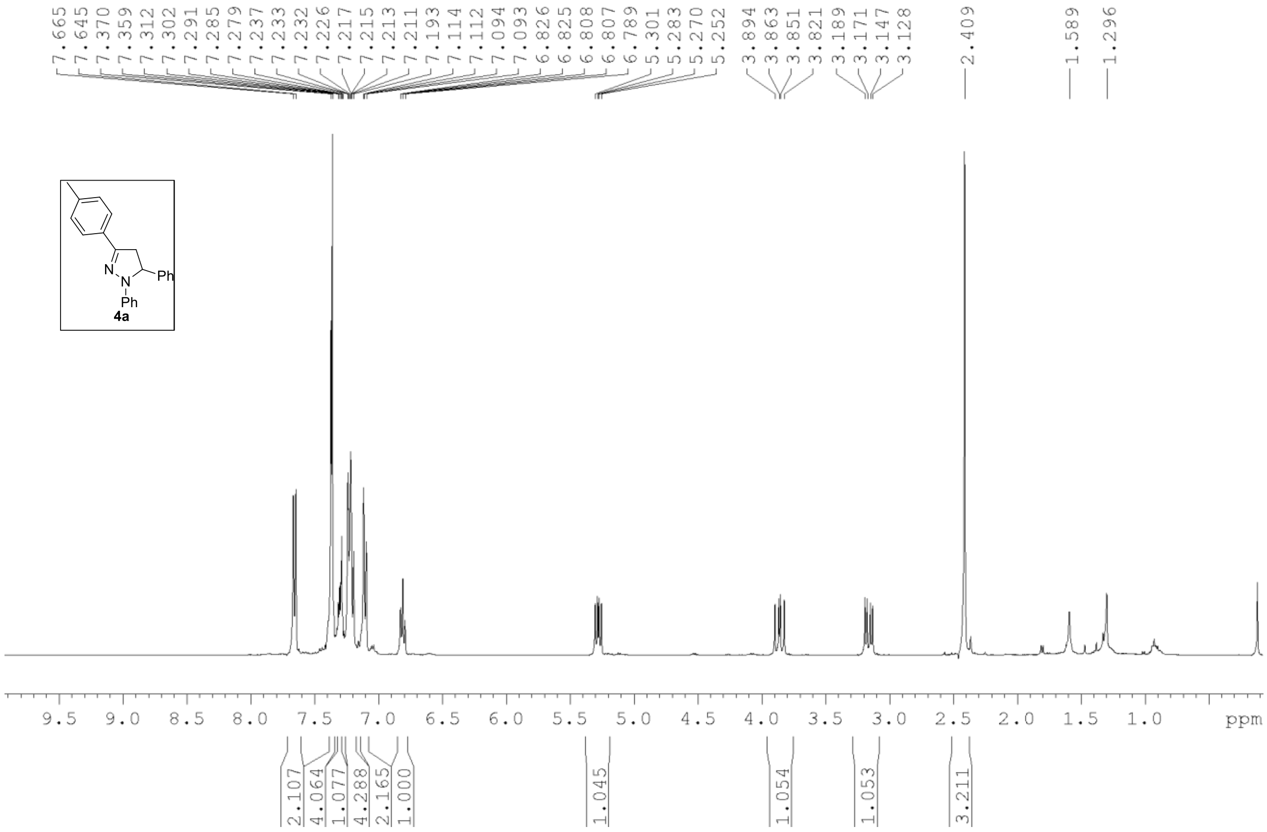

7.665 7.645 7.370 7.359 7.312 7.302 7.291 7.285 7.279 7.237 7.233 7.232 7.226 7.217 7.215 7.213 7.211 7.193 7.114 7.112 7.094 7.093 6.826 6.825 6.808 6.807 6.789 5.301 5.283 5.270 5.252 3.894 3.863 3.851 3.821 3.189 3.171 3.147 3.128 2.409 1.589 1.296
N
N
Ph
Ph
4a
9.5 9.0 8.5 8.0 7.5 7.0 6.5 6.0 5.5 5.0 4.5 4.0 3.5 3.0 2.5 2.0 1.5 1.0 ppm
2.107 4.064 1.077 4.288 2.165 1.000 1.045 1.054 1.053 3.211

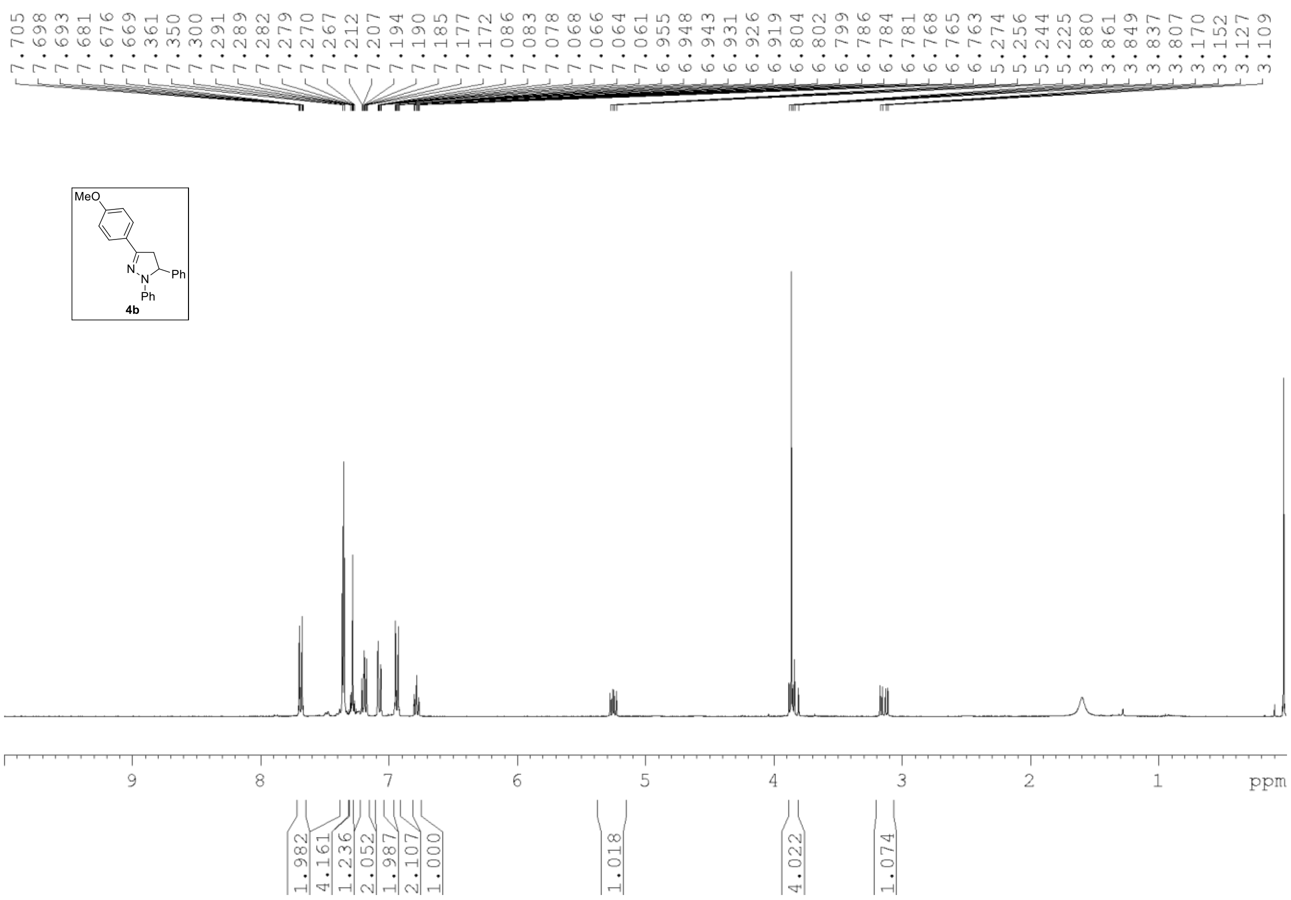

7.705 7.698 7.693 7.681 7.676 7.669 7.361 7.350 7.300 7.291 7.289 7.282 7.279 7.270 7.267 7.212 7.207 7.194 7.190 7.185 7.177 7.172 7.086 7.083 7.078 7.068 7.066 7.064 7.061 6.955 6.948 6.943 6.931 6.926 6.919 6.804 6.802 6.799 6.786 6.784 6.781 6.768 6.765 6.763 5.274 5.256 5.244 5.225 3.880 3.861 3.849 3.837 3.807 3.170 3.152 3.127 3.109
MeO
N
N
Ph
Ph
4b
9 8 7 6 5 4 3 2 1 ppm
1.982 4.161 1.236 2.052 1.987 2.107 1.000 1.018 4.022 1.074

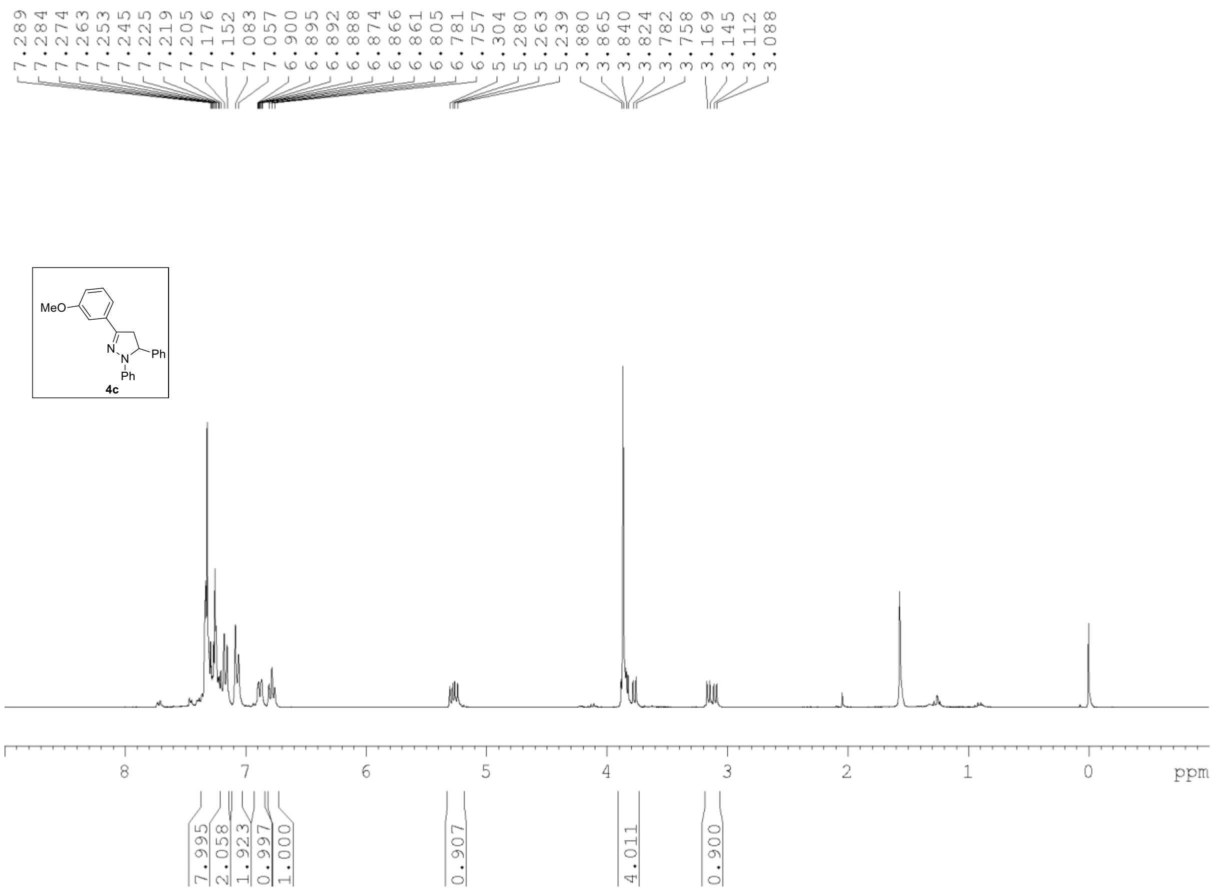
7.289
7.284
7.274
7.263
7.253
7.245
7.225
7.219
7.205
7.176
7.152
7.083
7.057
6.900
6.895
6.892
6.888
6.874
6.866
6.861
6.805
6.781
6.757
5.304
5.280
5.263
5.239
3.880
3.865
3.840
3.824
3.782
3.758
3.169
3.145
3.112
3.088
MeO
N
N
Ph
Ph
4c
8
7
6
5
4
3
2
1
0
ppm
7.995
2.058
1.923
0.997
1.000
0.907
4.011
0.900

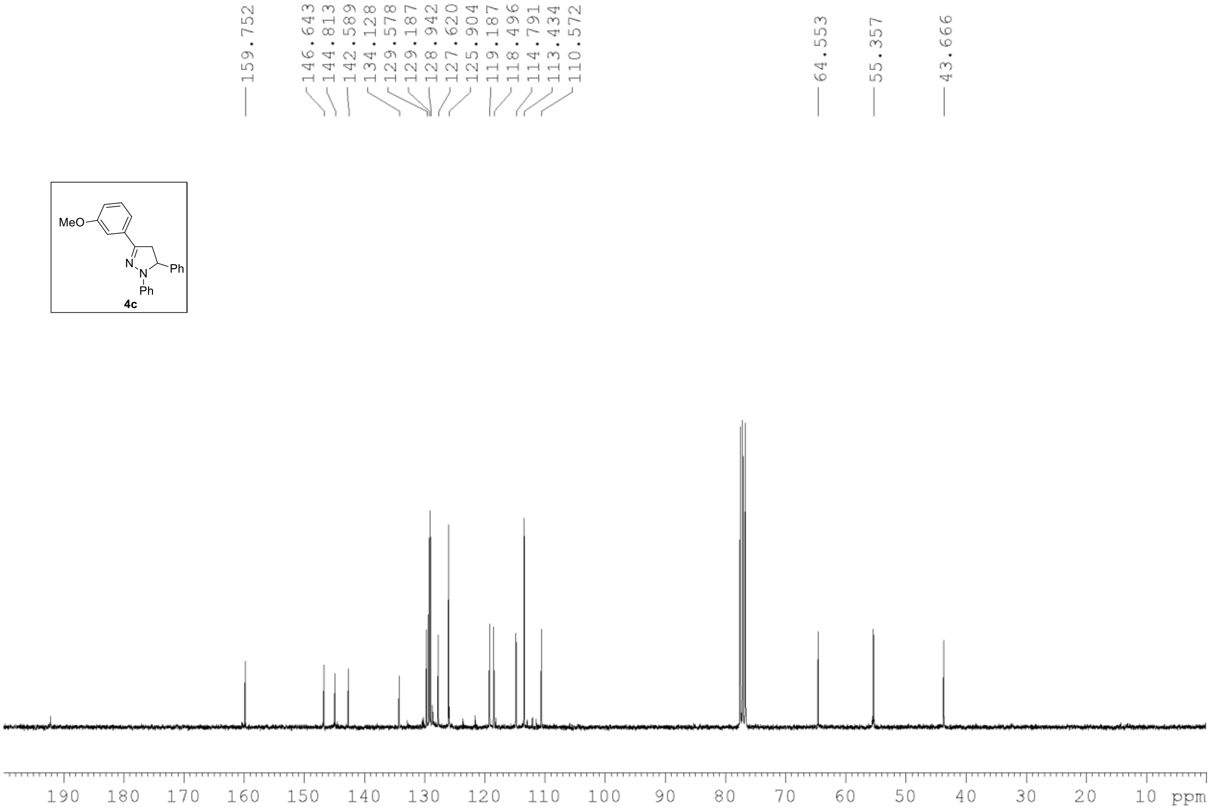
159.752
146.643
144.813
142.589
134.128
129.578
129.187
128.942
127.620
125.904
119.187
118.496
114.791
113.434
110.572
64.553
55.357
43.666
MeO
N
N
Ph
Ph
4c
190
180
170
160
150
140
130
120
110
100
90
80
70
60
50
40
30
20
10
ppm

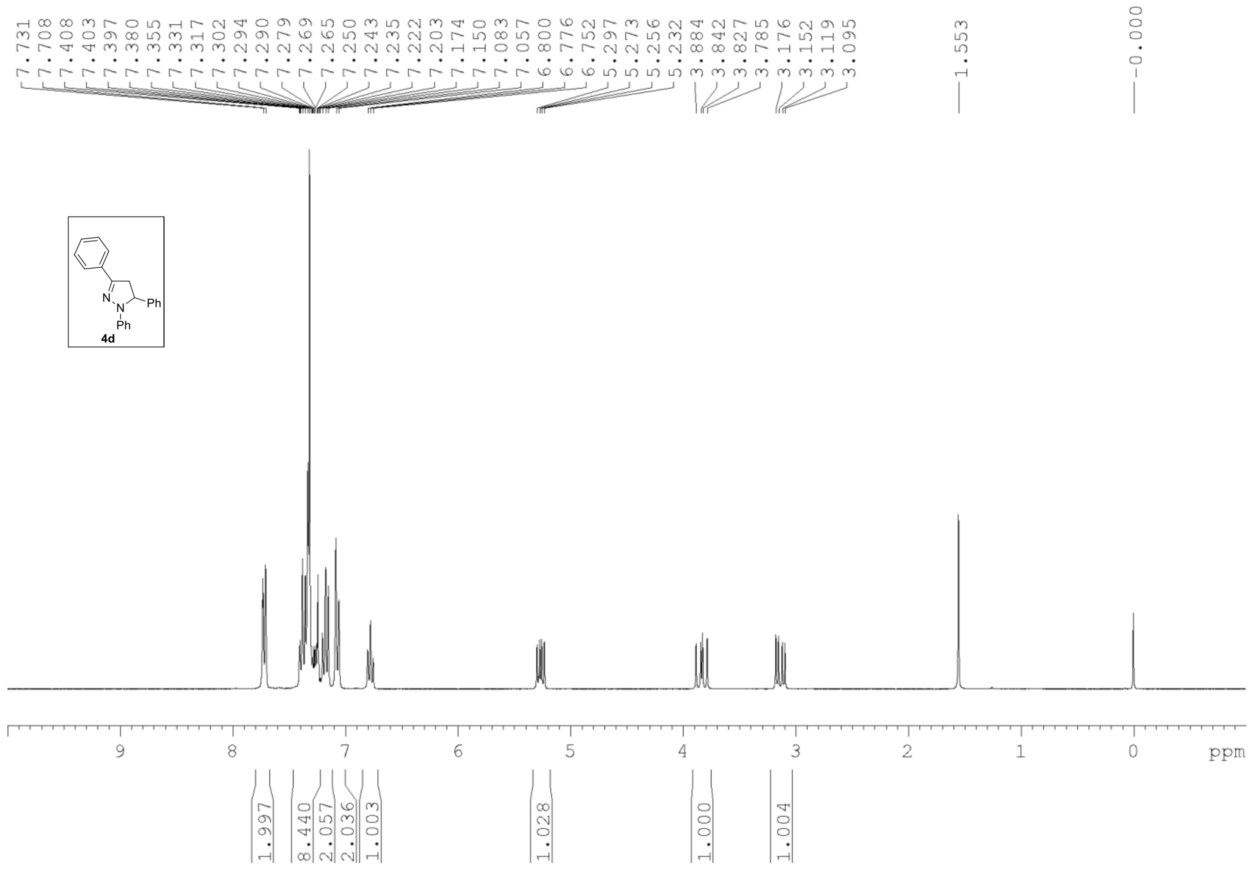
7.731
7.708
7.408
7.403
7.397
7.380
7.355
7.331
7.317
7.302
7.294
7.290
7.279
7.269
7.265
7.250
7.243
7.235
7.222
7.203
7.174
7.150
7.083
7.057
6.800
6.776
6.752
5.297
5.273
5.256
5.232
3.884
3.842
3.827
3.785
3.176
3.152
3.119
3.095
1.553
0.000
N
N
Ph
Ph
4d
9
8
7
6
5
4
3
2
1
0
ppm
1.997
8.440
2.057
2.036
1.003
1.028
1.000
1.004

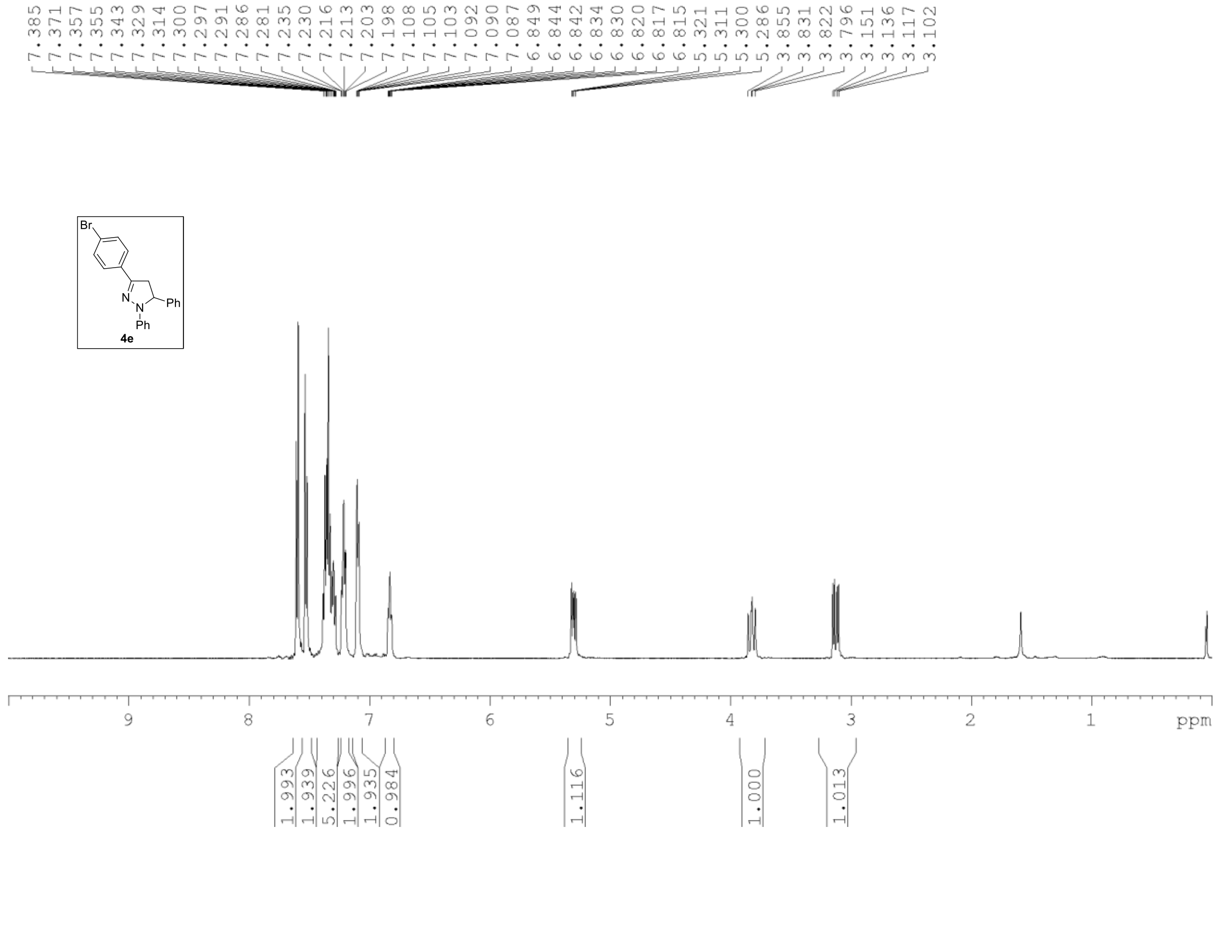
7.385
7.371
7.357
7.355
7.343
7.329
7.314
7.300
7.297
7.291
7.286
7.281
7.235
7.230
7.216
7.213
7.203
7.198
7.108
7.105
7.103
7.092
7.090
7.087
6.849
6.842
6.834
6.830
6.820
6.817
6.815
5.321
5.311
5.300
5.286
3.855
3.831
3.822
3.796
3.151
3.136
3.117
3.102
Br
N
N
Ph
Ph
4e
9
8
7
6
5
4
3
2
1
ppm
1.993
1.939
5.226
1.996
1.935
0.984
1.116
1.000
1.013

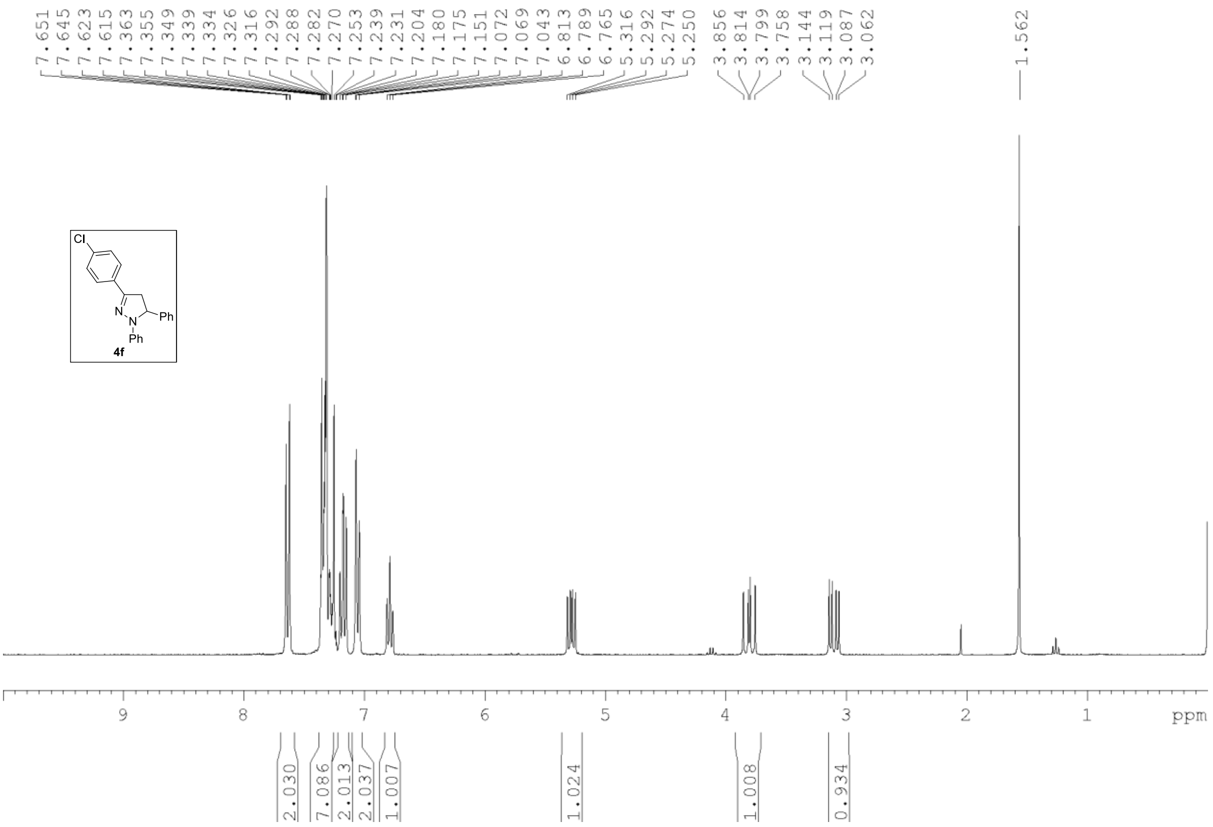
7.651 7.645 7.623 7.615 7.363 7.355 7.349 7.339 7.334 7.326 7.316 7.292 7.288 7.282 7.270 7.253 7.239 7.231 7.204 7.180 7.175 7.151 7.072 7.069 7.043 6.813 6.789 6.765 5.316 5.292 5.274 5.250 3.856 3.814 3.799 3.758 3.144 3.119 3.087 3.062 1.562
Cl
N
N
Ph
Ph
4f
9 8 7 6 5 4 3 2 1 ppm
2.030 7.086 2.013 2.037 1.007 1.024 1.008 0.934

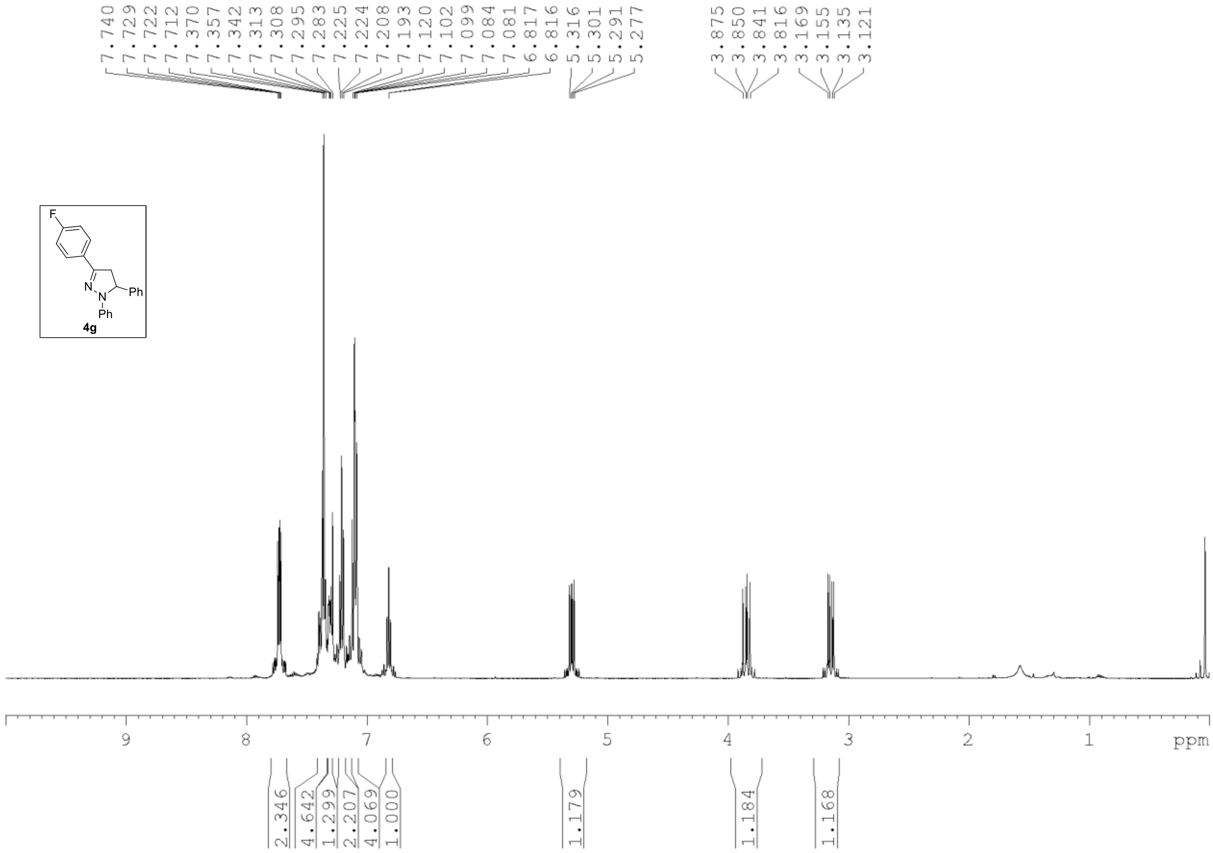
7.740 7.729 7.722 7.712 7.370 7.357 7.342 7.313 7.308 7.295 7.283 7.225 7.224 7.208 7.193 7.120 7.102 7.099 7.084 7.081 6.817 6.816 5.316 5.301 5.291 5.277 3.875 3.850 3.841 3.816 3.169 3.155 3.135 3.121
F
N
N
Ph
Ph
4g
9 8 7 6 5 4 3 2 1 ppm
2.346 4.642 1.299 2.207 4.069 1.000 1.179 1.184 1.168

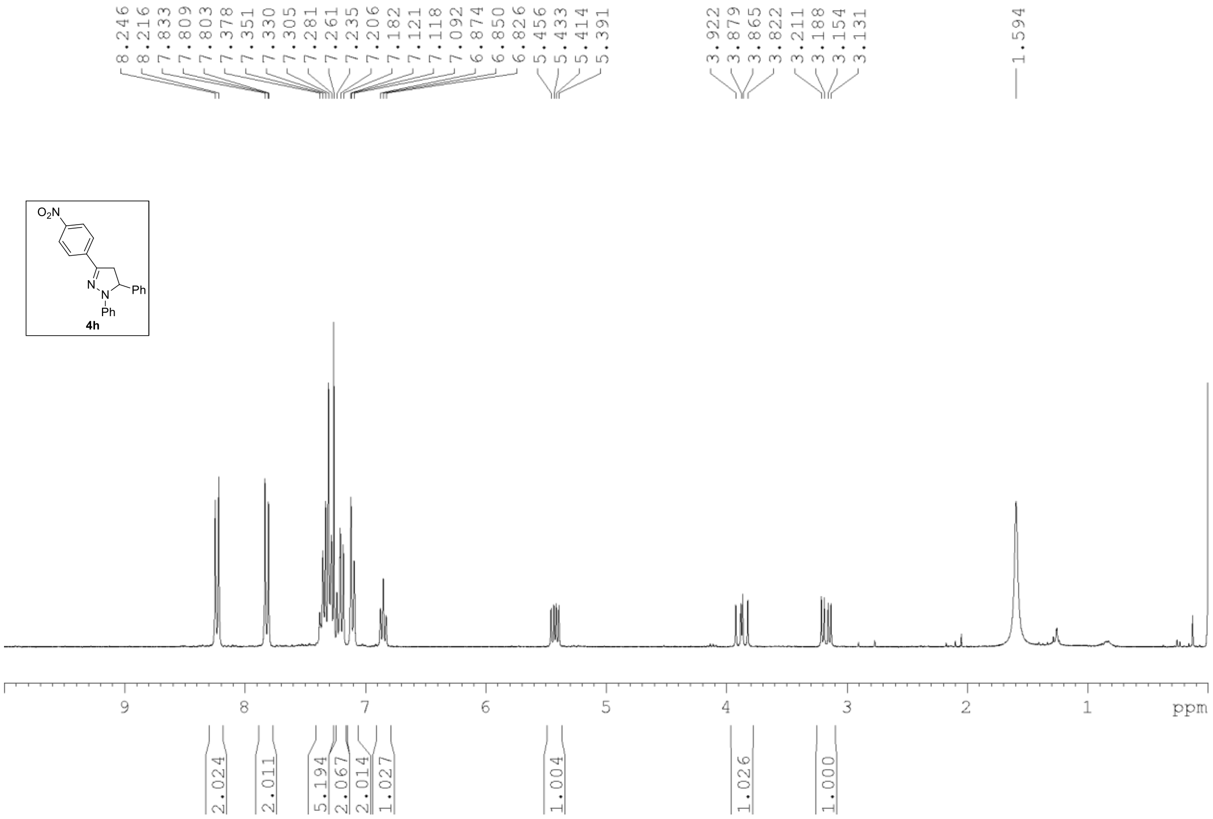

8.246
8.216
7.833
7.809
7.803
7.378
7.351
7.330
7.305
7.281
7.261
7.235
7.206
7.182
7.121
7.118
7.092
6.874
6.850
6.826
5.456
5.433
5.414
5.391
3.922
3.879
3.865
3.822
3.211
3.188
3.154
3.131
1.594
O_2N
N
N
Ph
Ph
4h
9 8 7 6 5 4 3 2 1 ppm
2.024
2.011
5.194
2.067
2.014
1.027
1.004
1.026
1.000

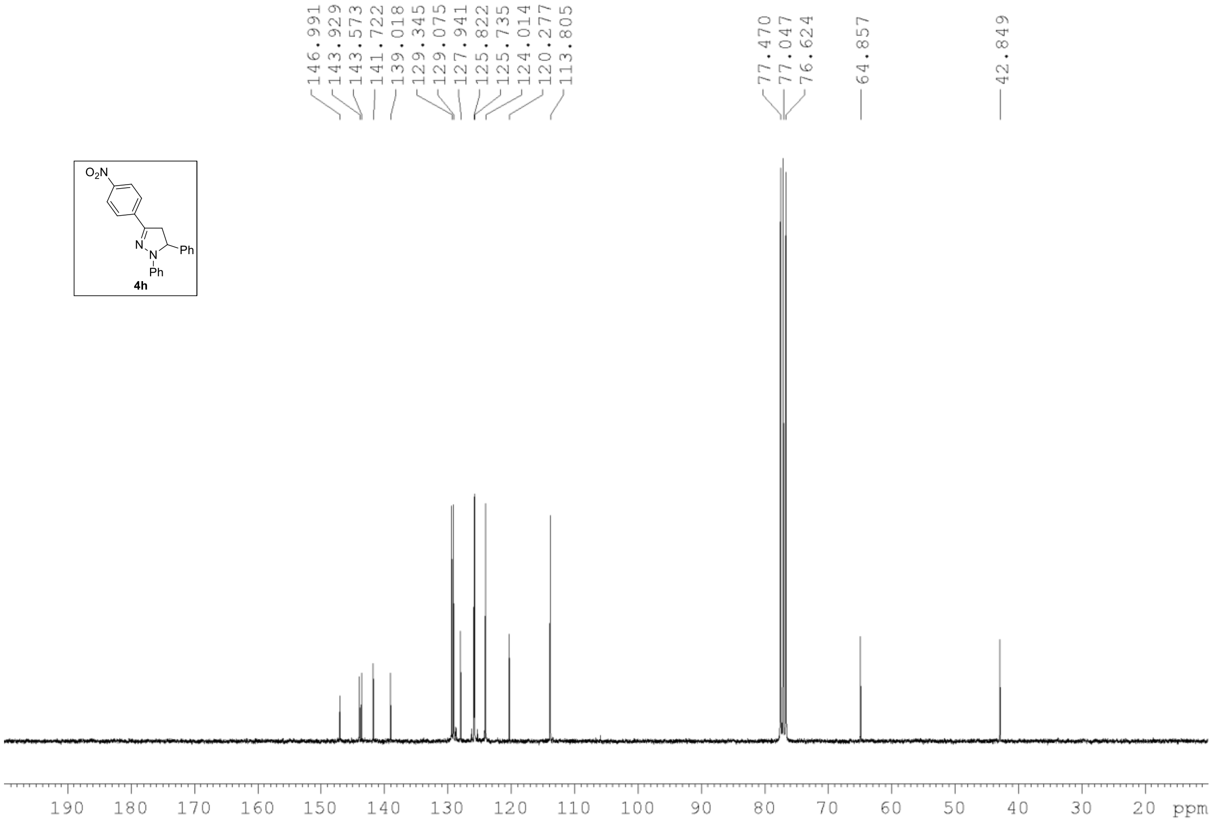

146.991
143.929
143.573
141.722
139.018
129.345
129.075
127.941
125.822
125.735
124.014
120.277
113.805
77.470
77.047
76.624
64.857
42.849
O_2N
N
N
Ph
Ph
4h
190 180 170 160 150 140 130 120 110 100 90 80 70 60 50 40 30 20 ppm

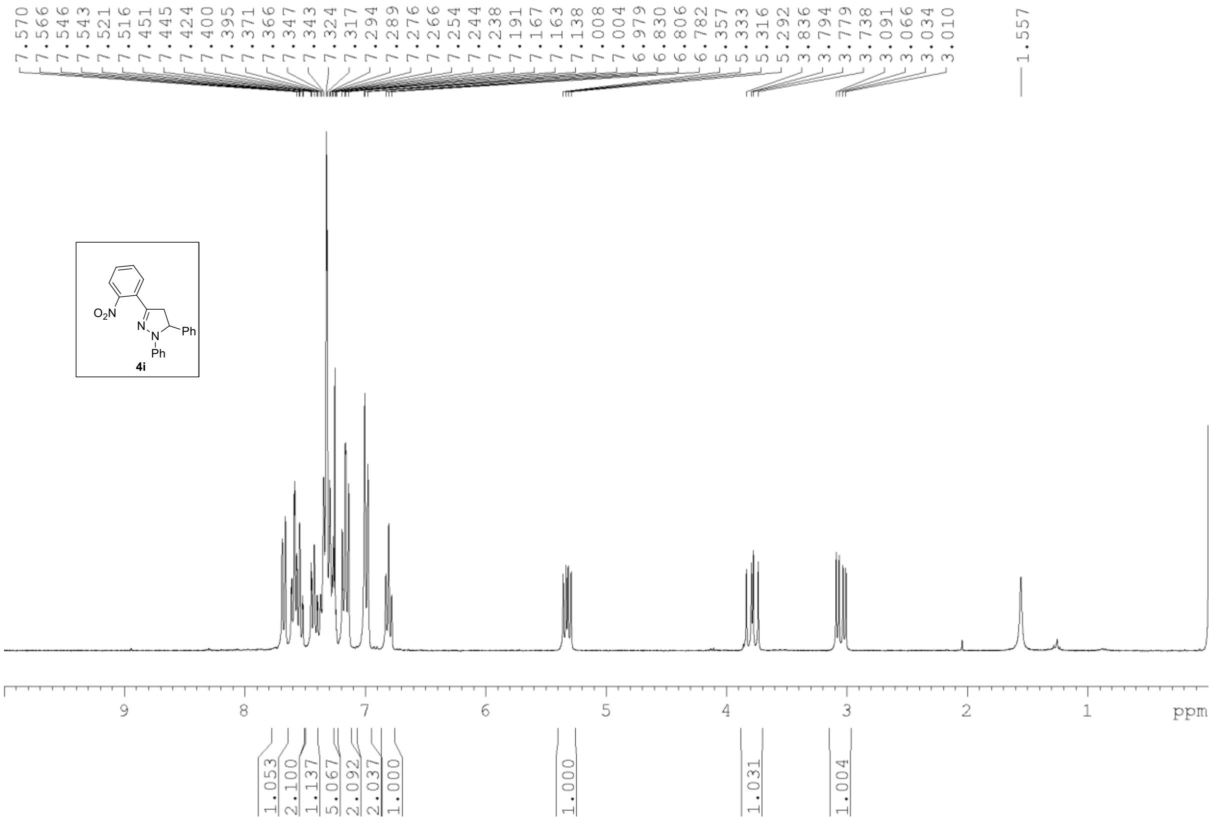
7.570
7.566
7.546
7.543
7.521
7.516
7.451
7.445
7.424
7.400
7.395
7.371
7.366
7.347
7.343
7.324
7.317
7.294
7.289
7.276
7.266
7.254
7.244
7.238
7.191
7.167
7.163
7.138
7.008
7.004
6.979
6.830
6.806
6.782
5.357
5.333
5.316
5.292
3.836
3.794
3.779
3.738
3.091
3.066
3.034
3.010
1.557
O2N
N
N
Ph
Ph
4i
ppm
1.053
2.100
1.137
5.067
2.092
2.037
1.000
1.000
1.031
1.004

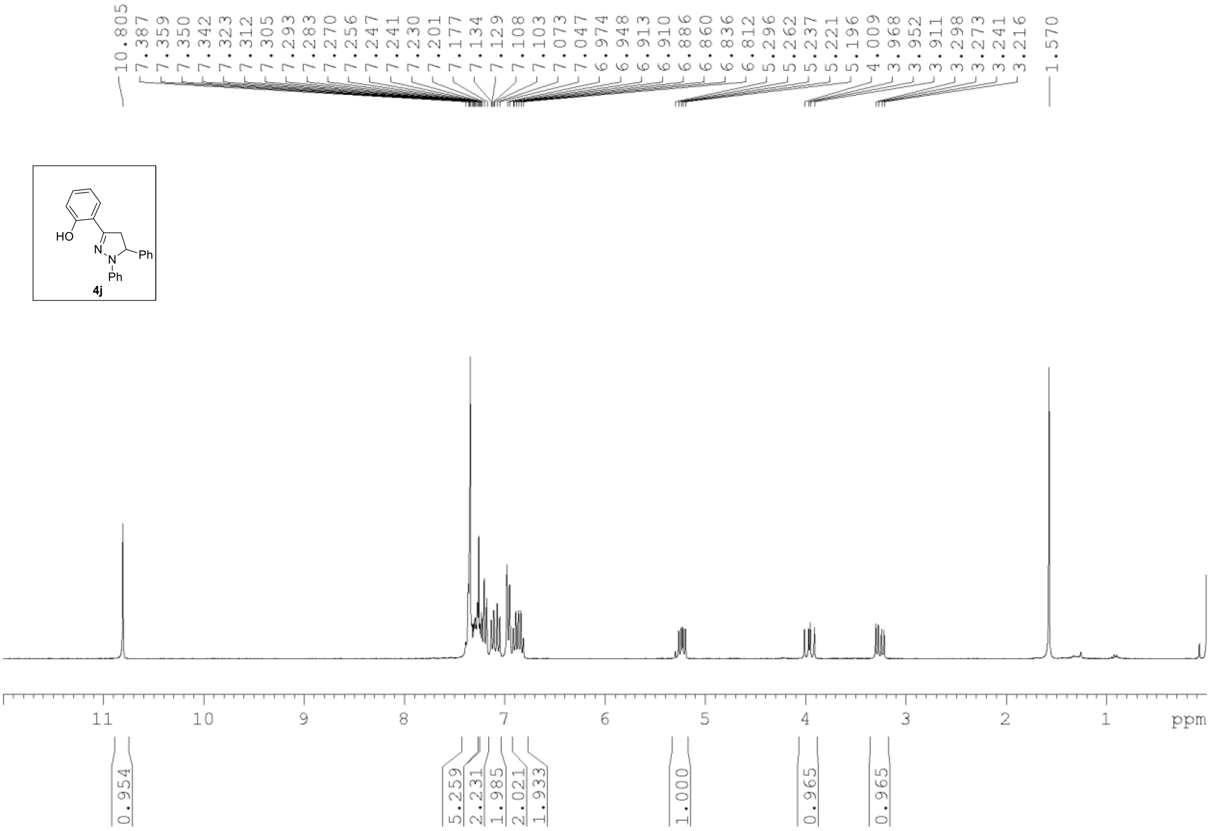
10.805
7.387
7.359
7.350
7.342
7.323
7.312
7.305
7.293
7.283
7.270
7.256
7.247
7.241
7.230
7.201
7.177
7.134
7.129
7.108
7.103
7.073
7.047
6.974
6.948
6.913
6.910
6.886
6.860
6.836
6.812
5.296
5.262
5.237
5.221
5.196
4.009
3.968
3.952
3.911
3.298
3.273
3.241
3.216
1.570
HO
N
N
Ph
Ph
4j
ppm
0.954
5.259
2.231
1.985
2.021
1.933
1.000
0.965
0.965

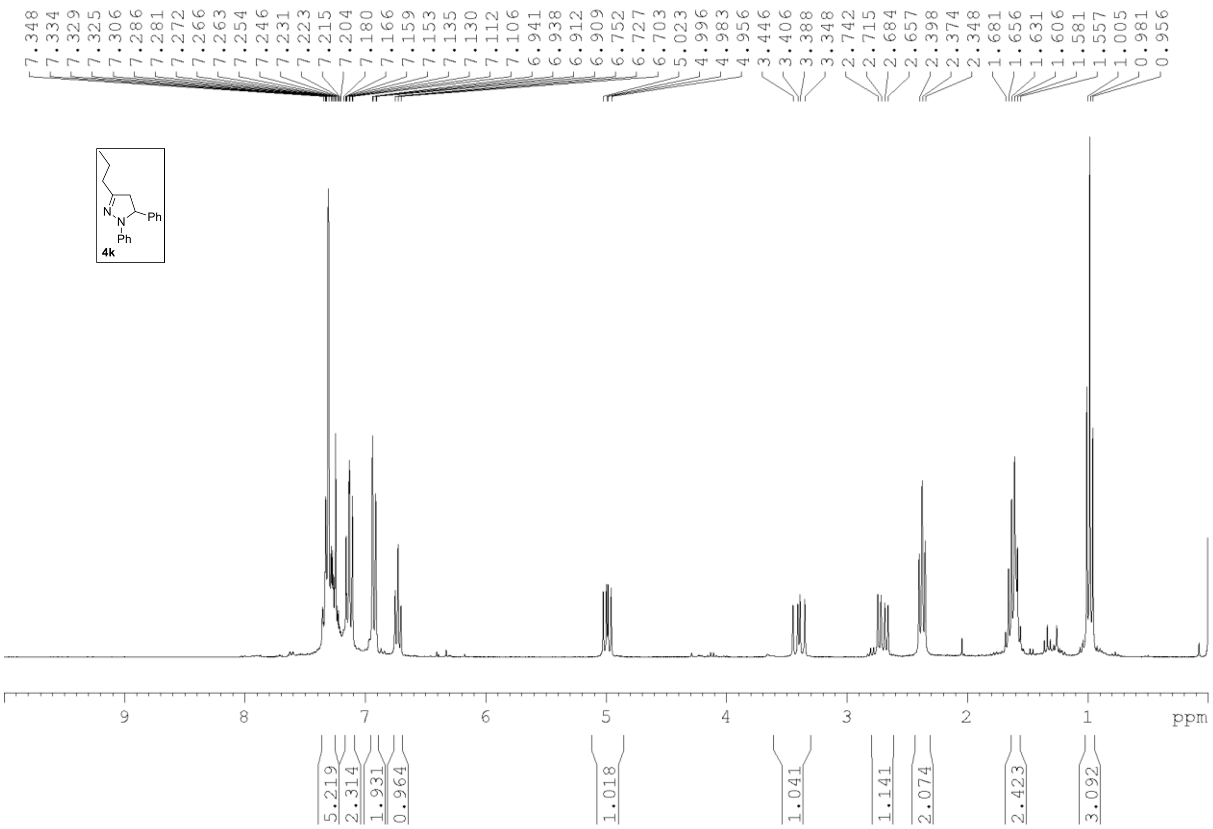
7.348
7.334
7.329
7.325
7.306
7.286
7.281
7.272
7.266
7.263
7.254
7.246
7.231
7.223
7.215
7.204
7.180
7.166
7.159
7.153
7.135
7.130
7.112
7.106
6.941
6.938
6.912
6.909
6.752
6.727
6.703
5.023
4.996
4.983
4.956
3.446
3.406
3.388
3.348
2.742
2.715
2.684
2.657
2.398
2.374
2.348
1.681
1.656
1.631
1.606
1.581
1.557
1.005
0.981
0.956
N
N
Ph
Ph
4k
9
8
7
6
5
4
3
2
1
ppm
5.219
2.314
1.931
0.964
1.018
1.041
1.141
2.074
2.423
3.092

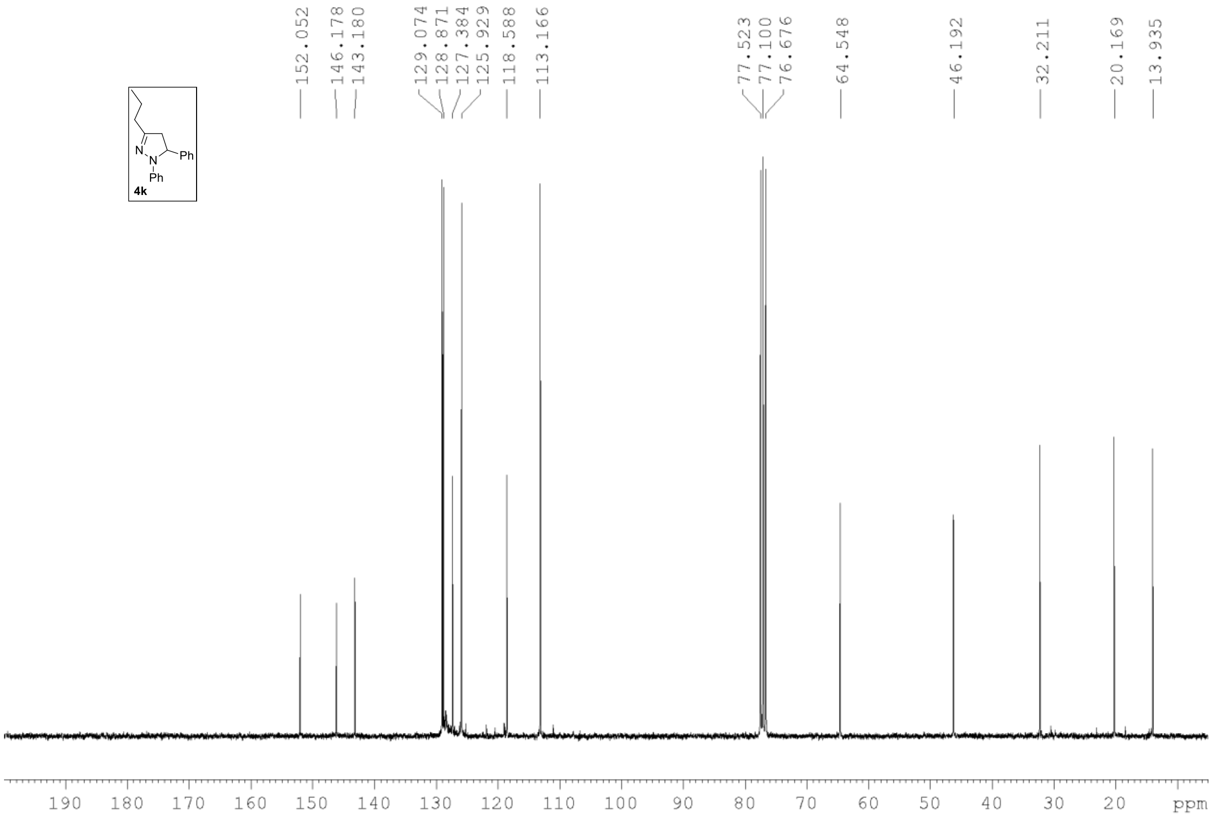
152.052
146.178
143.180
129.074
128.871
127.384
125.929
118.588
113.166
77.523
77.100
76.676
64.548
46.192
32.211
20.169
13.935
N
N
Ph
Ph
4k
190
180
170
160
150
140
130
120
110
100
90
80
70
60
50
40
30
20
ppm

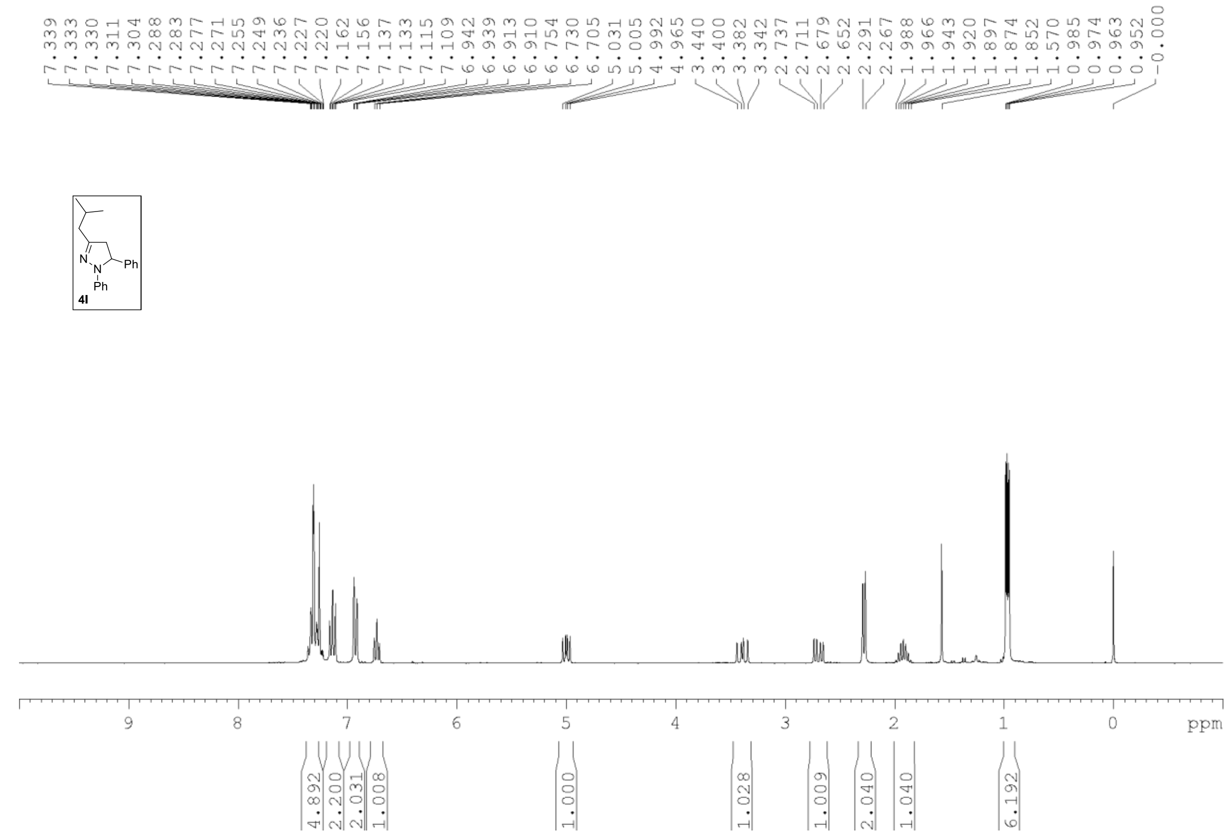
7.339
7.333
7.330
7.311
7.304
7.288
7.283
7.277
7.271
7.255
7.249
7.236
7.227
7.220
7.162
7.156
7.137
7.133
7.115
7.109
6.942
6.939
6.913
6.910
6.754
6.730
6.705
5.031
5.005
4.992
4.965
3.440
3.400
3.382
3.342
2.737
2.711
2.679
2.652
2.291
2.267
1.988
1.966
1.943
1.920
1.897
1.874
1.852
1.570
0.985
0.974
0.963
0.952
-0.000
N
N
Ph
Ph
4l
9
8
7
6
5
4
3
2
1
0
ppm
4.892
2.200
2.031
1.008
1.000
1.028
1.009
2.040
1.040
6.192

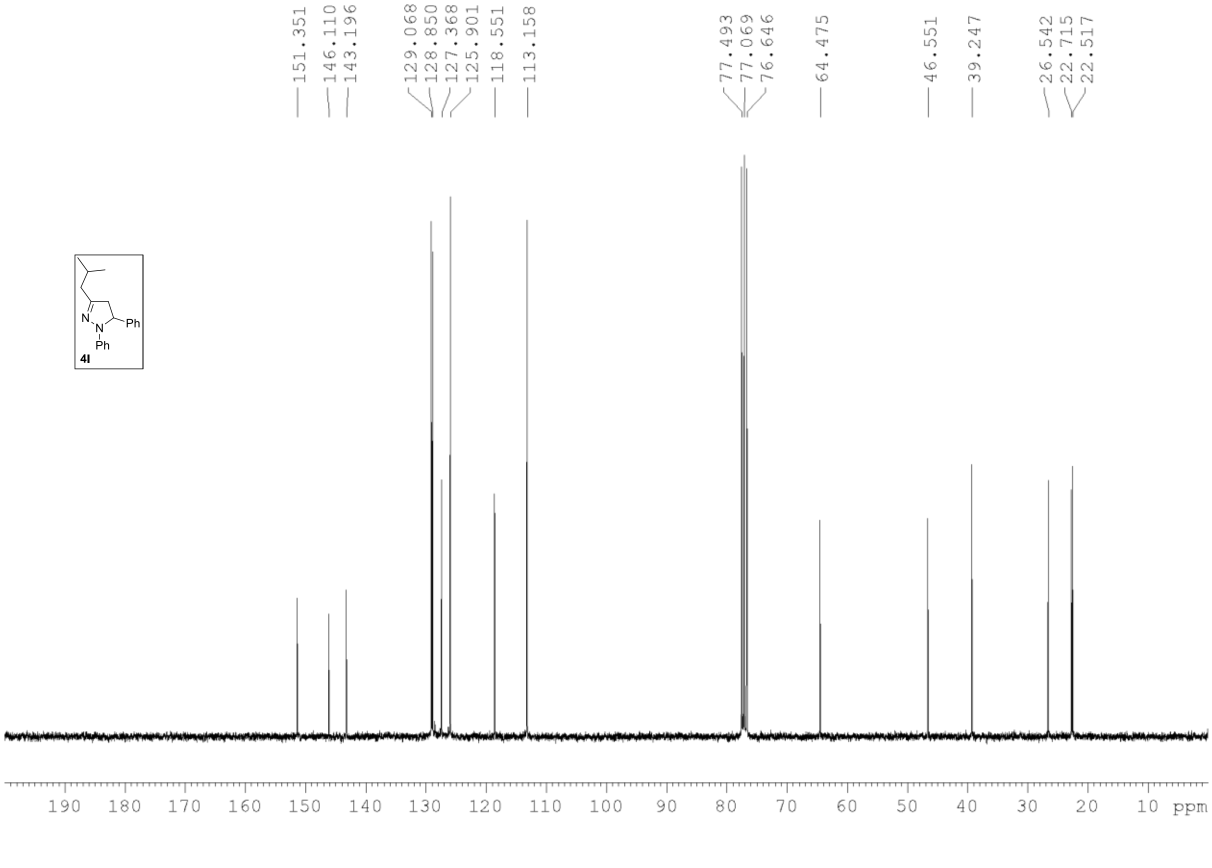
151.351
146.110
143.196
129.068
128.850
127.368
125.901
118.551
113.158
77.493
77.069
76.646
64.475
46.551
39.247
26.542
22.715
22.517
N
N
Ph
Ph
4l
190
180
170
160
150
140
130
120
110
100
90
80
70
60
50
40
30
20
10
ppm

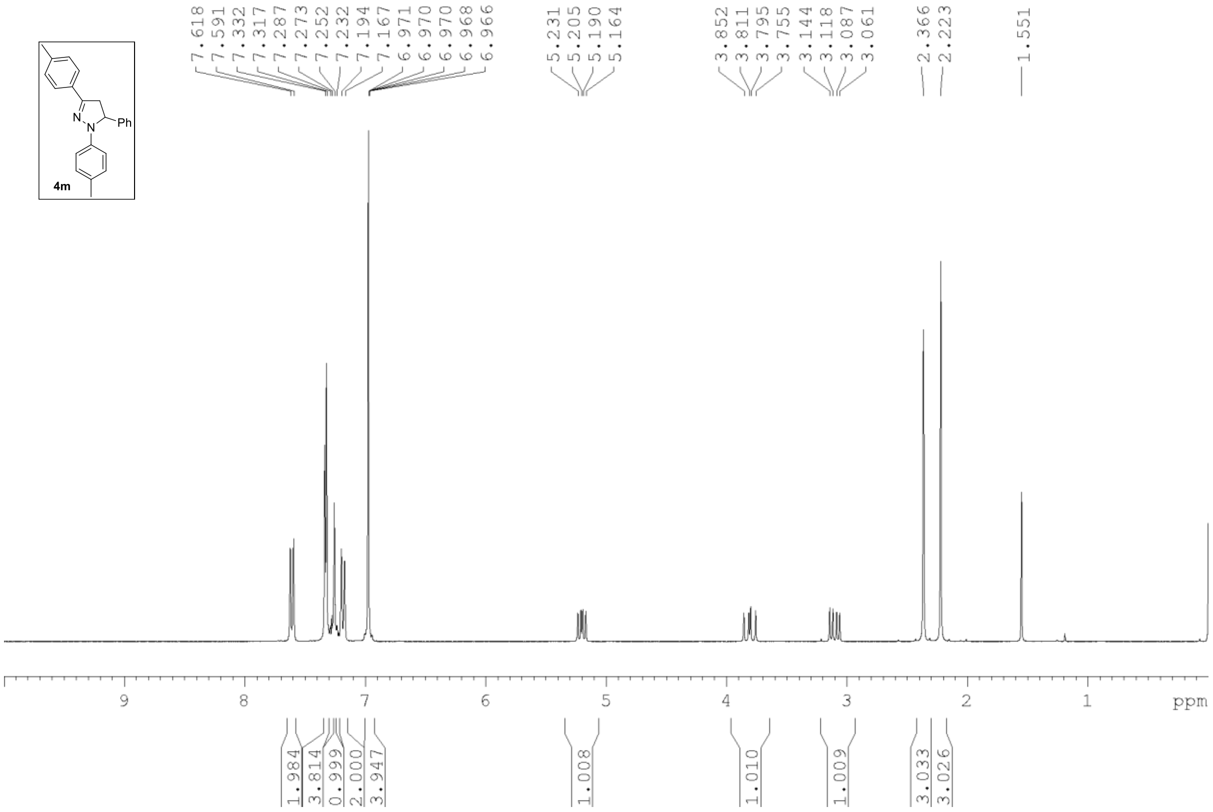
4m
Ph
N
7.618 7.591 7.332 7.317 7.287 7.273 7.252 7.232 7.194 7.167 6.971 6.970 6.970 6.968 6.966
5.231 5.205 5.190 5.164
3.852 3.811 3.795 3.755 3.144 3.118 3.087 3.061
2.366 2.223
1.551
9 8 7 6 5 4 3 2 1 ppm
1.984 3.814 0.999 2.000 3.947
1.008
1.010
1.009
3.033 3.026

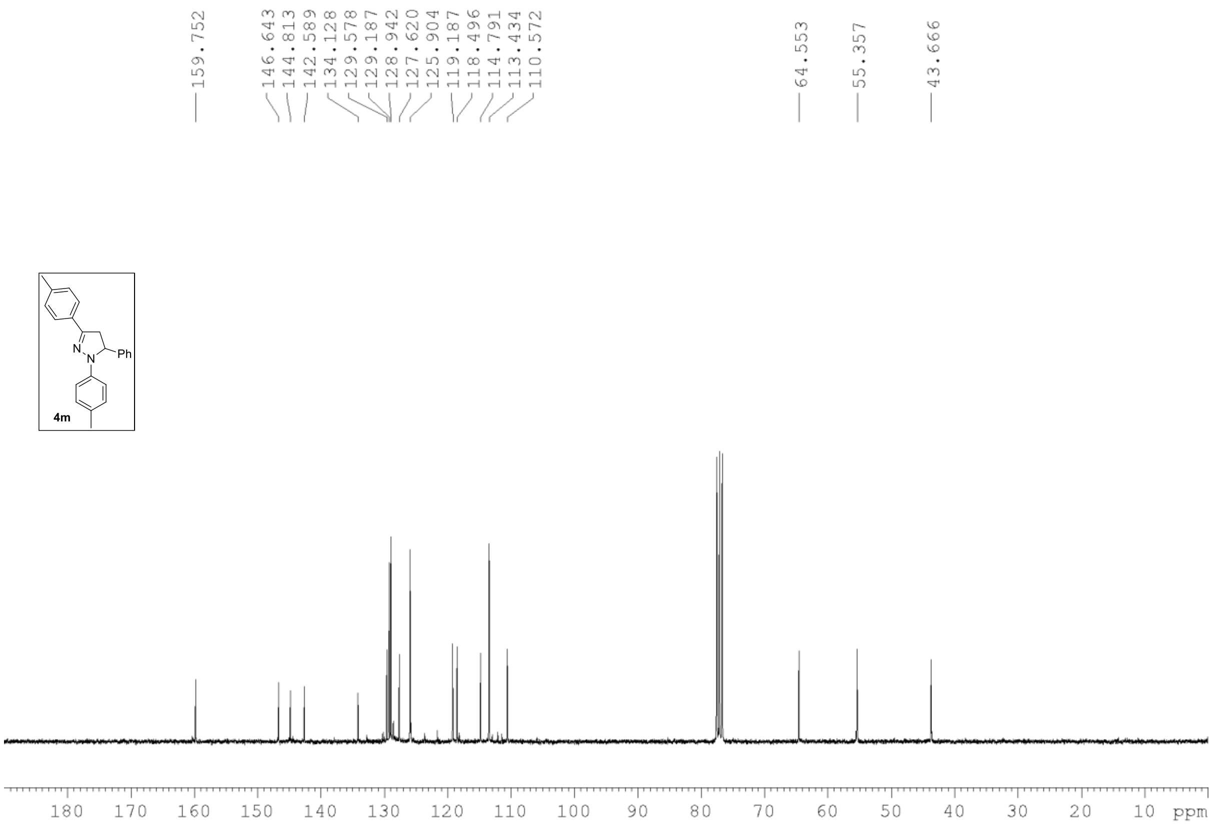
4m
Ph
N
159.752
146.643 144.813 142.589 134.128 129.578 129.187 128.942 127.620 125.904 119.187 118.496 114.791 113.434 110.572
64.553
55.357
43.666
180 170 160 150 140 130 120 110 100 90 80 70 60 50 40 30 20 10 ppm

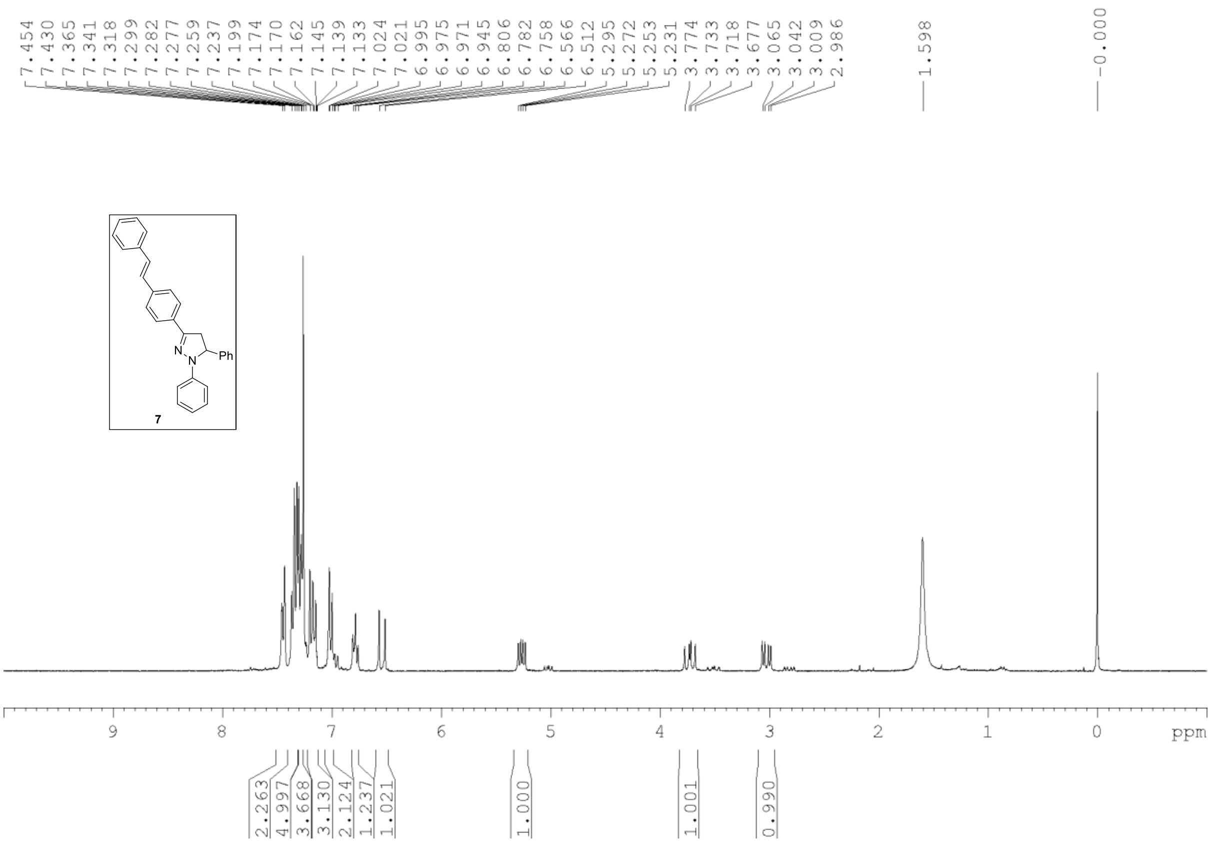
7.454 7.430 7.365 7.341 7.318 7.299 7.282 7.277 7.259 7.237 7.199 7.174 7.170 7.162 7.145 7.139 7.133 7.024 7.021 6.995 6.975 6.971 6.945 6.806 6.782 6.758 6.566 6.512 5.295 5.272 5.253 5.231 3.774 3.733 3.718 3.677 3.065 3.042 3.009 2.986
1.598
-0.000
Ph
N
N
7
9 8 7 6 5 4 3 2 1 0 ppm
2.263 4.997 3.668 3.130 2.124 1.237 1.021
1.000
1.001
0.990

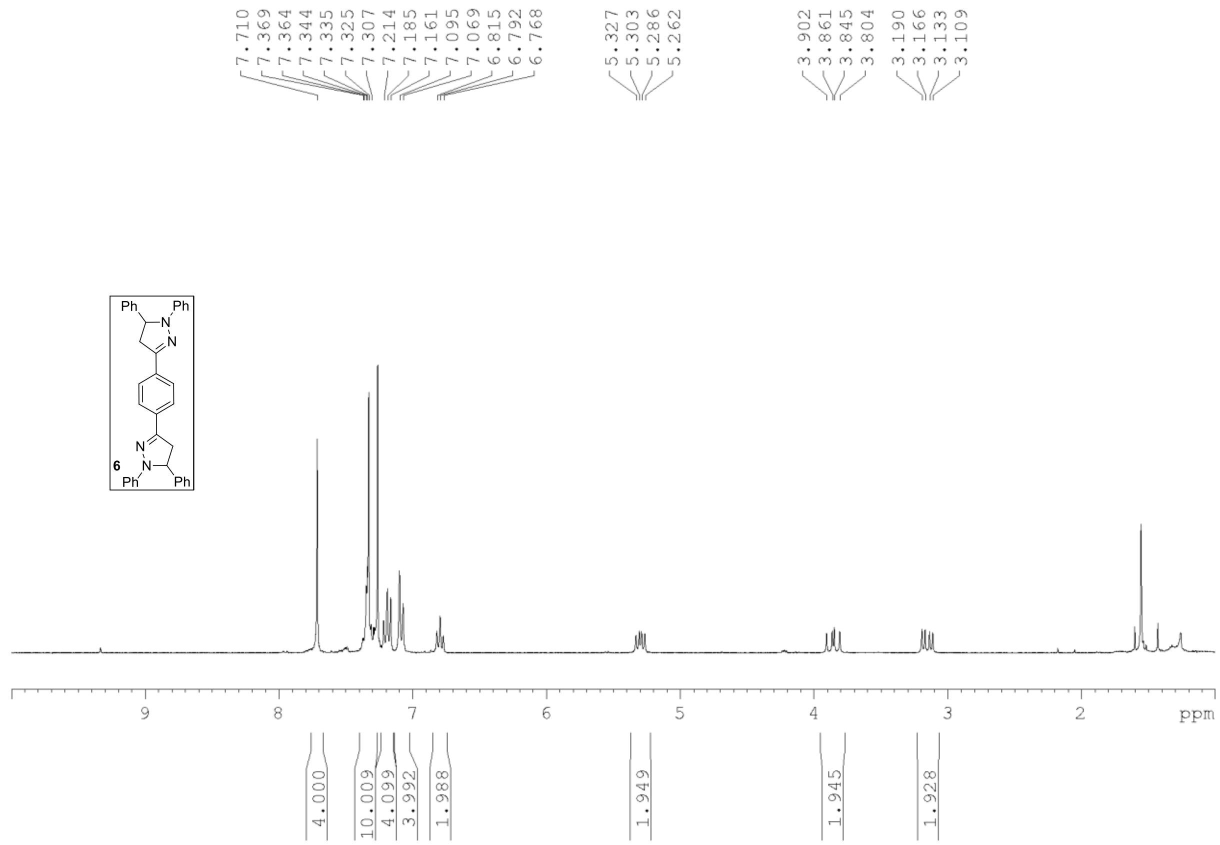
7.710 7.369 7.364 7.344 7.335 7.325 7.307 7.214 7.185 7.161 7.095 7.069 6.815 6.792 6.768
5.327 5.303 5.286 5.262
3.902 3.861 3.845 3.804
3.190 3.166 3.133 3.109
Ph Ph N N
N N
6
Ph Ph
9 8 7 6 5 4 3 2 ppm
4.000
10.009 4.099 3.992 1.988
1.949
1.945
1.928
